# Establishment of a CRISPR/Cas9 knockout library for screening type I interferon-inducible antiviral effectors in pig cells

**DOI:** 10.3389/fimmu.2022.1016545

**Published:** 2022-11-24

**Authors:** Wen Dang, Tao Li, Fan Xu, Yannan Wang, Fan Yang, Haixue Zheng

**Affiliations:** ^1^ State Key Laboratory of Veterinary Etiological Biology, College of Veterinary Medicine, Lanzhou University, Lanzhou Veterinary Research Institute, Chinese Academy of Agricultural Sciences, Lanzhou, China; ^2^ Lanzhou University Second Hospital, Department of Radiology, Lanzhou, China

**Keywords:** interferons, interferon-stimulated genes, CRISPR/Cas9, VSV-eGFP, IBRS-2

## Abstract

Diseases caused by emerging swine viruses had a great economic impact, constituting a new challenge for researchers and practicing veterinarians. Innate immune control of viral pathogen invasion is mediated by interferons (IFNs), resulting in transcriptional elevation of hundreds of IFN-stimulated genes (ISGs). However, the ISG family is vast and species-specific, and despite remarkable advancements in uncovering the breadth of IFN-induced gene expression in mouse and human, it is less characterized with respect to the repertoire of porcine ISGs and their functional annotation. Herein, with the application of RNA-sequencing (RNA-Seq) gene profiling, the breadth of IFN-induced gene expression in the context of type I IFN stimulation was explored by using IBRS-2 cell, a commonly used high-efficient cultivation system for porcine picornaviruses. By establishing inclusion criteria, a total of 359 ISGs were selected. Aiming to identify key effectors mediating type I IFN inhibition of swine viruses, a CRISPR/Cas9 knockout library of 1908 sgRNAs targeting 5’ constitutive exons of 359 ISGs with an average of 5 to 6 sgRNAs per gene was constructed. Using VSV-eGFP (vesicular stomatitis virus, fused with GFP) as a model virus, a subset of highest-ranking candidates were identified, including previously validated anti-VSV genes IRF9, IFITM3, LOC100519082 and REC8, as well as several novel hits. This approach attains a high level of feasibility and reliability, and a high rate of hit identification, providing a forward-looking platform to systematically profile the effectors of type I IFN antiviral response against porcine viruses.

## Introduction

The type I interferon (IFN) response is at the frontline in defending cells from viral pathogen invasion (1). The activation of the IFN-triggered JAK-STAT pathway results in the expression of a diverse range of gene products that serve as ultimate effectors fighting against virus replication. The genes encoding for those cellular antiviral factors are called interferon-stimulated genes (ISGs). Although hundreds of ISGs have been identified, their functional annotation with respect to antiviral potential, target specificity and concurrent mechanism-of-action is poorly characterized (2–5). Thus, it is of great need and importance to identify and characterize sets of novel ISGs for each viral species susceptible to type I IFN treatment. The follow-up endeavors to better understand the exact mechanism-of-action of newly identified ISGs have unearthed a wide range of targets for rational development of therapeutic options against virus infection.

Of note, it is estimated that approximately 10% of the genes in the human genome have the potential to be regulated by stimulation with IFNs. From decades of research, it is discovered that ISG products take on a diverse range of roles, including antiviral defense, anti-proliferative activities, and stimulation of adaptive immunity. Even though great strides into deciphering the antiviral mechanism of a select group of classic ISGs have been made, advances in profiling novel sets of antiviral effectors towards each viral species, however, have largely been limited. Previously, remarkable progress has been made using gain-of-function screening in which single ISGs were expressed and their antiviral activity was assessed against different viruses. Using expressional screening of 29 and 39 ISGs, restriction factors including Viperin, ISG20 and PKR have been identified for HCV (6), as well as IFI27, IRG1 and Viperin for neurotropic RNA viruses (7). In 2011, large-scale, fluoresce-activated, cell sorting-based overexpression screening of 389 human ISGs identified a diverse range of gene products as effectors of type I IFN antiviral response against six important human and animal viruses (8). However, gain-of-function screens are labor-intensive and clearly incapable of characterizing all relevant ISGs. Instead, loss-of-function can overcome these limitations. Loss-of-function genome-wide screen based on RNA interference (RNAi) libraries is temporarily used in identifying antiviral IFN effectors against HCV, however, drawbacks of RNAi libraries such as off-target effect hinder its wide application (9). Very recently, through genome-scale CRISPR loss-of-function screening, IFI6 was identified as an ER-resident interferon effector that blocked flavivirus replication, further potentializing CRISPR/Cas9 approach as a validated tool for screening IFN effectors (10).

To date, few studies were dedicated to examining the repertoire of ISGs in the porcine species. With the application of RNA sequencing (RNA-Seq) gene profiling, a total of 359 cellular effectors were selected as ISGs and their expression kinetics in the context of type I IFN stimulation were characterized in Instituto Biologico-Rim Suino-2 (IBRS-2) cells. Existing evidence demonstrated that IBRS-2 possessed susceptibility to the viruses of foot-and-mouth disease, swine vesicular disease, vesicular exanthema of swine, transmissible gastroenteritis, and several other viruses (11). Meanwhile, IBRS-2 cells have an aberrant RIG-I-like receptor (RLR) pathway but an intact type I IFN pathway, which facilitates the characterization of downstream antiviral effectors in the context of virus infection and IFN stimulation (12). To this aim, we constructed a CRISPR/Cas9 knockout library of 1809 unique sequences targeting 359 ISGs with an average coverage of 5 to 6 sgRNAs per gene in IBRS-2 cells. Following lentiviral delivery in IBRS-2 cells, positive selection screening identified interferon effectors crucial for blocking VSV-eGFP (Vesicular Stomatitis Virus (VSV)) replication. The highest ranking candidates included previously validated anti-VSV genes IRF9, LOC100519082 (also referred to as IFITM1), IFITM3 and REC8, as well as novel hits. This ISG-targeting CRISPR/Cas9 knockout library demonstrated a high level of consistency between type I IFNs (including two subtypes of interferon α and one subtype of interferon β) and a high rate of hit confirmation, demonstrating the promise of wide application in screening novel sets of ISGs against a panel of porcine viruses.

## Materials and methods

### Virus, cell lines and reagents

VSV-eGFP (Cat#: NTA-2011-ZP20; Creative Biolabs) stock was propagated in BHK-21 cell culture, titrated by 50% haemadsorbing doses (HAD_50_) assay, aliquoted and preserved at -80°C before use. IBRS-2 and HEK 293T cells were cultured in Dulbeccos Modified Eagle Medium (DMEM) (GIBCO, Invitrogen China Limited, Shanghai, China) supplemented with 10% (v/v) fetal bovine serum (FBS) (BI, Biological Industries Israel Beit Haemek LTD., Kibbutz Beit-Haemek, Israel) and 1% (v/v) penicillin-streptomycin solution (100 I.U./mL penicillin, 5 mg/L streptomycin) (Gibco).

Recombinant human IFN-α 1b (Cat#: Z02866), IFN-α 2a (Cat#: Z03003) and IFN-β (Cat#: Z03109) were purchased from GenScript (Nanjing, China), reconstituted in PBS containing 0.1% BSA to a concentration of 10 μg/mL, and preserved at -80°C before use. Ruxolitinib (RUX) (CAS No.: 941678-49-5) (Cat#: HY-50856) was obtained from MedChemExpress (MCE), stocked at 10 mM in DMSO and preserved at -80°C before use.

### RNA extraction, library preparation and sequencing

IBRS-2 cells were seeded into a 6-well plate at 1 × 10^6^ cells per well. Following overnight culture, the plate was replenished with fresh growth medium containing 10 ng/mL of type I IFNs. At the indicated time points, cell monolayers were rinsed with pre-cold PBS three times, and total RNA was extracted using the mirVana™ miRNA Isolation Kit (Ambion-1561) as per the manufacturer’s protocol. RNA concentration was measured by UV spectrophotometry (NanoDrop™ 2000) and the RNA integrity was assessed using the Agilent 2100 Bioanalyzer System (Agilent Technologies, Santa Clara, CA, USA). The samples could be subjected to further experiment only if RNA integrity number (RIN) was greater than or equal to 7. The libraries were constructed using reagents and protocols supplied in TruSeq Stranded mRNA LTSample Prep Kit (Cat#: RS-122-2101) (Illumina, San Diego, CA, USA). Then these libraries were validated by BioAnalyzer followed by 125-bp/150-bp paired-end read sequencing on the Illumina sequencing platform (HiSeq^®^ 2500 Sequencing Systems and HiSeq X™ Ten), as carried out by OE Biotech Co., Ltd. (Shanghai, China).

### Sequence analysis

Following quality control [processing using Trimmomatic (13), removal of adapter sequences and trimming], clean reads were mapped to Sscrofa11.1 using HISAT2 with default parameters (14). Mapped reads were subsequently assembled into transcripts, with FPKM values and read counts being calculated by Cufflinks (15) and HTSeq (16), respectively. Transcript abundance was compared between mock and type I IFN-treated samples using the DESeq (version 1.24.0) R package to identify differentially expressed genes (DEGs). Inclusion criteria of a *p* value of < 0.05, a twofold or greater expression change and a FPKM value greater than 1 in at least one sample was set as the threshold for DEGs.

### Library construction

Oligos encoding the sgRNA library with ~1908 specific sgRNA sequences targeting 359 ISGs were synthesized by a programmable microarray using the Synthesizer (GenScript, Wuhan) and cloned as a pool into lentiCRISPRv2 vector. Library quality was assessed by NGS with coverage of 100%. Following lentiviral production, IBRS-2 cells were infected with the lentiCRISPRv2-derived lentivirus at low titer (MOI = 0.05) and high titer (MOI = 0.1) for 24 h, respectively, and reseeded into growth medium containing 5 μg/mL of puromycin. Following three consecutive rounds of selective culture, approximately 4 days per round, transduced cells were subjected to further characterization of transduction efficacy.

### CRISPR screening

Briefly, puromycin-selected IBRS-2 cell populations were infected with VSV-eGFP at MOI of 0.1, followed by treatment with type I IFNs for 36 h. The eGFP-positive cells that were no longer sensitive to type I IFN-mediated inhibition were harvested by fluorescent activated cell sorting (FACS). Genomic DNA was extracted from those cells, and sgRNA sequences were amplified by PCR and deep sequenced.

### Lentiviral production and transduction

In order to overexpress selected top four ISGs in IBRS-2 cells, a bicistronic lentiviral vector co-expressing an ISG and the red fluorescent protein, TagRFP was constructed. Control vector co-expressing GFP/TagRFP was used in the present study. Lentiviral particles were produced by co-transfecting HEK293T cells with plasmids expressing: 1) the ISG/TagRFP proviral DNA, 2) HIV gap-pol, and 3) the vesicular stomatitis virus glycoprotein (VSV-G) in a ratio of 1:0.8:0.2, respectively. Following 6 h transfection a media change to DMEM with 3% FBS was performed. Supernatants were harvested at 48 h and 72 h, pooled, clarified by centrifugation, and stored at −80°C before use.

IBRS-2 cells were seeded into a 12 well plate at a density of 5 × 10^5^ cells per well. Following overnight culture, cells were transduced with lentiviral particles at an MOI of 0.5. After 24 h incubation the cells were subjected to fluorescent microscopy for assessing the transduction efficiency.

### VSV-eGFP infection

In order to determine the effects of top four ISGs on viral replication, vector control and ISG/TagRFP-expressing IBRS-2 cells were inoculated with VSV-eGFP at an MOI of 0.1. At 48 h post inoculation, the whole culture was freezed at −80°C and subsequently thawed at 37°C. After repeated freezing and thawing, the culture was clarified by ultracentrifugation and viral titers were measured by TCID_50_ assay.

## Results

### VSV-eGFP is highly sensitive to type I IFN treatment

**Figure 1:**
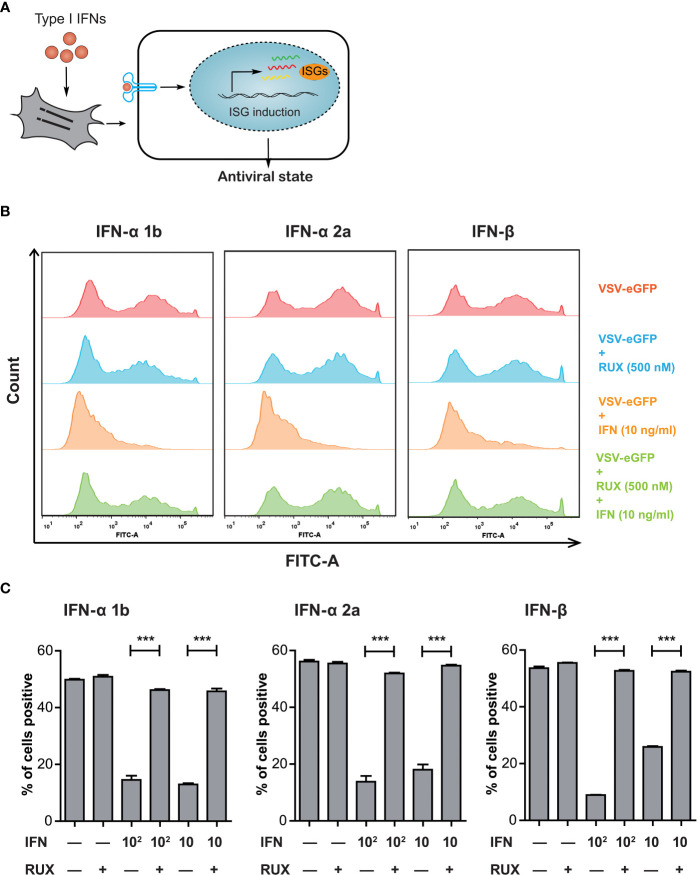
VSV-eGFP possessed a sensitivity to type I IFN-triggered antiviral response. **(A)** Treatment with exogenous type I IFNs induces the expression of hundreds of ISGs, allowing the establishment of a so-called antiviral state against pathogen invasion in host cells. **(B)** Representative Flow cytometry analysis of EGFP expression rate in VSV-eGFP-infected IBRS-2 cells mock-treated (top), treated with RUX (500 nM) (middle, upper) and IFN (10 ng/mL) (middle, lower) alone or in combination (bottom). Cells without infection were tested as a background fluorescence intensity control (not shown in the histogram). VSV-eGFP replication was effectively inhibited by type I IFN treatment but subsequently restored by supplementation of RUX. **(C)** Graphs showing Flow cytometry data analysis indicating the reversion of IFN inhibition by addition of RUX. Data are expressed as mean ± SEM. ***P < 0.001.

The IFN-mediated induction of hundreds of ISGs confers host cells an antiviral state against pathogen invasion (**Figure 1A**). The VSV replication is highly sensitive to type I IFN-induced antiviral responses (17, 18). In this regard, IBRS-2 cells pre-infected with VSV-eGFP at an MOI of 0.1 were subsequently treated with varying concentration of type I IFNs for 24 h. Virus replication was directly monitored as eGFP-positive cells by flow cytometry. As indicated, type I IFNs including IFN-α 1b, IFN-α 2a and IFN-β exerted antiviral activity against VSV-eGFP replication at concentrations of 10 and 100 ng/mL (**Figures 1B, C**). To identify the association between type I IFN-mediated inhibition and activation of the JAK-STAT pathway, VSV-eGFP-infected IBRS-2 cells were incubated with type I IFNs in combination with Ruxolitinib, a well-known JAK1 and JAK2 inhibitor. Of note, the addition of Ruxolitinib at 500 nM abolished the antiviral potential of type I IFNs and concurrently restored the VSV-eGFP replication (**Figures 1B, C**). Collectively, VSV-eGFP possessed sensitivity to treatment with type I IFNs. Pharmacological blocking of the JAK-STAT pathway abolished IFN inhibition and reversed VSV-eGFP replication.

### RNA-Seq of type I IFN-treated IBRS-2 cells identified a list of 359 ISGs

**Figure 2:**
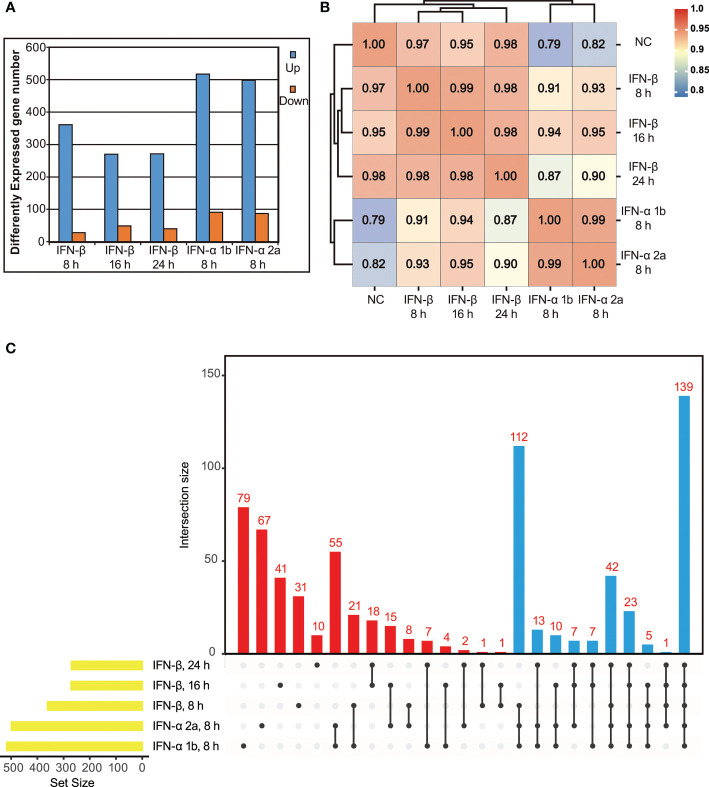
Identification of ISGs induced by exogenous type I IFNs using RNA-Seq. **(A)** Differentially expressed genes (*p* value < 0.05, twofold or more change and FPKM value greater than 1 in at least one sample) for each IFN-treated sample are depicted numerically. **(B)** Correlation matrix of all 5 IFN-treated samples (based on Pearson correlation coefficients). **(C)** Upset plot of up-regulated DEGs in IFN-treated samples. Of note, all the up-regulated DEGs in the blue bars are clustered into ISG family.

**Figure 3:**
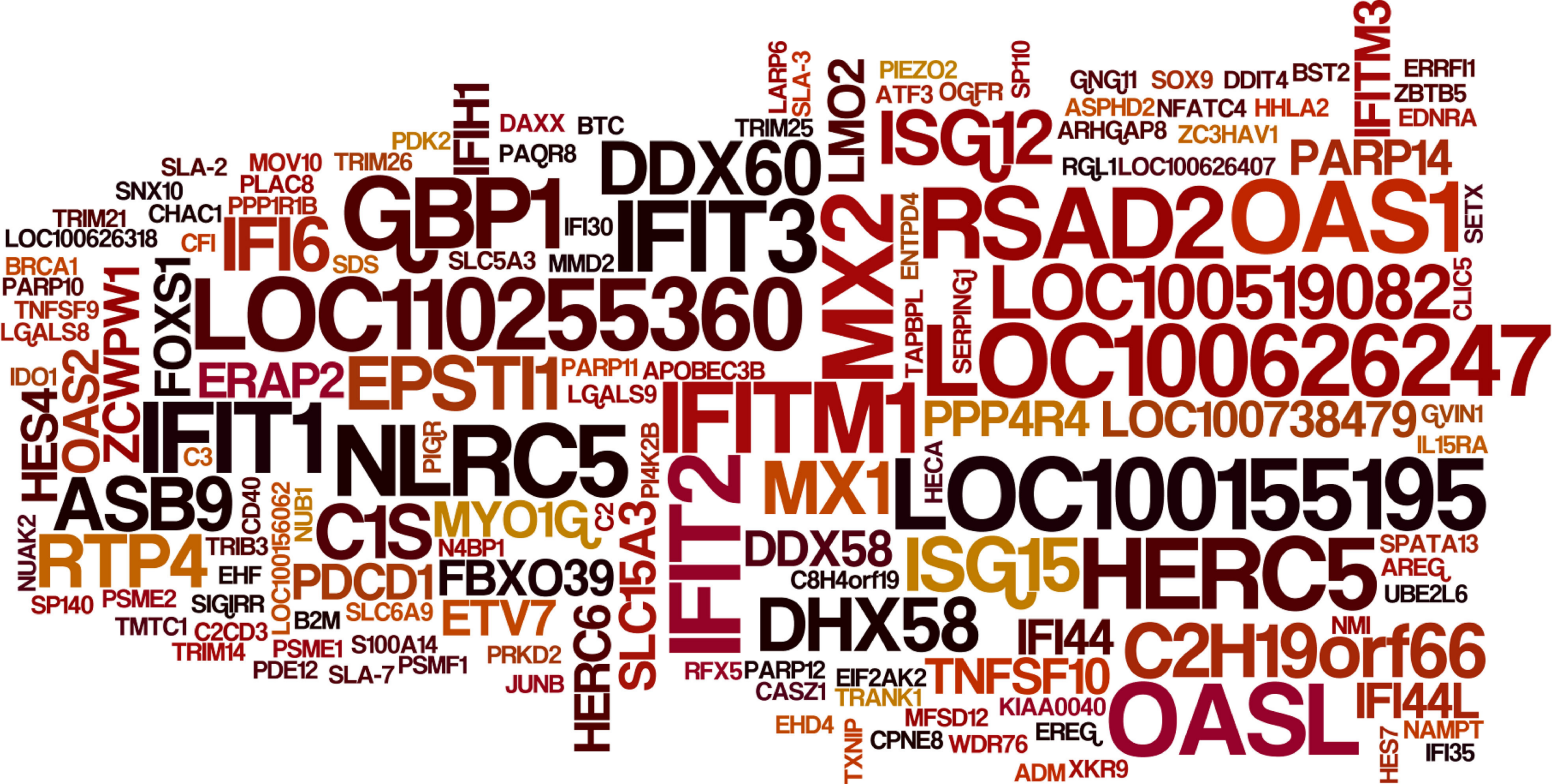
Expression levels of select ISGs in type I IFN-treated IBRS-2 cells. The font size of each ISG is directly proportional to its average fold change in type I IFN-treated IBRS-2 cells normalized to mock-treated cells. Of note, ISGs with biggest font size demonstrated highest expression levels with fold changes greater than 1000.

To conduct a large-scale antiviral ISG screening, the transcriptional response to type I IFNs in IBRS-2 cells was analyzed by using RNA-Seq and inclusion criteria was established. To evaluate the kinetics of ISG expression reached by type I IFNs, a preliminary experiment was performed by challenging IBRS-2 cells with IFN-β (10 ng/mL) for 8 h, 16 h and 24 h. A simple enumeration of DEGs revealed that up-regulated DEGs achieved maximal amount by 8 h, began to decline thereafter, and reached a plateau at 16 h post treatment (**Figure 2A**). Conversely, several genes were significantly down-regulated over time. Given the fact that IFN-β stimulated the most up-regulated DEGs at the very early stage of treatment, an 8 h duration is reasonably regarded as the optimal incubation time for IFNs to trigger the most ISGs in IBRS-2 cells. Thus, fresh IBRS-2 cells were stimulated with IFN-α 1b (10 ng/mL) or IFN-α 2a (10 ng/mL) singly for 8 h, and subjected to RNA-Seq. Of significant note is that IFN-α 1b and IFN-α 2a were capable of inducing more up-regulated DEGs, totaling 519 and 499, respectively (**Figure 2A**). In addition, the pearson correlation analysis visualizing the correlation (r) values revealed a significant positive association, suggestive of a consistent pattern of gene expression among type I IFN-treated samples, albeit the fact that transcriptional response to IFN-α 1b and IFN-α 2a was more analogous (**Figure 2B**). As demonstrated by upset plot analysis, noticeable is the fact that up-regulated DEGs induced by type I IFNs overlapped considerably but were also slightly specialized, with 79 DEGs unique to IFN-α 1b, 67 DEGs unique to IFN-α 2a, and 82 DEGs unique to IFN-β (**Figure 2C**). Considering that among hundreds of DEGs some were not specifically induced in response to type I IFN treatment, a DEG was qualified for the ISG family only if it was consistently up-regulated in at least three or more samples across all five samples (**Figure 2C**, blue bars). As such, a list of 359 ISGs was compiled in the present study (**Figure 3**, **Supplementary Table 1**).

### Construction and characterization of IBRS-2 knockout cell populations

**Figure 4:**
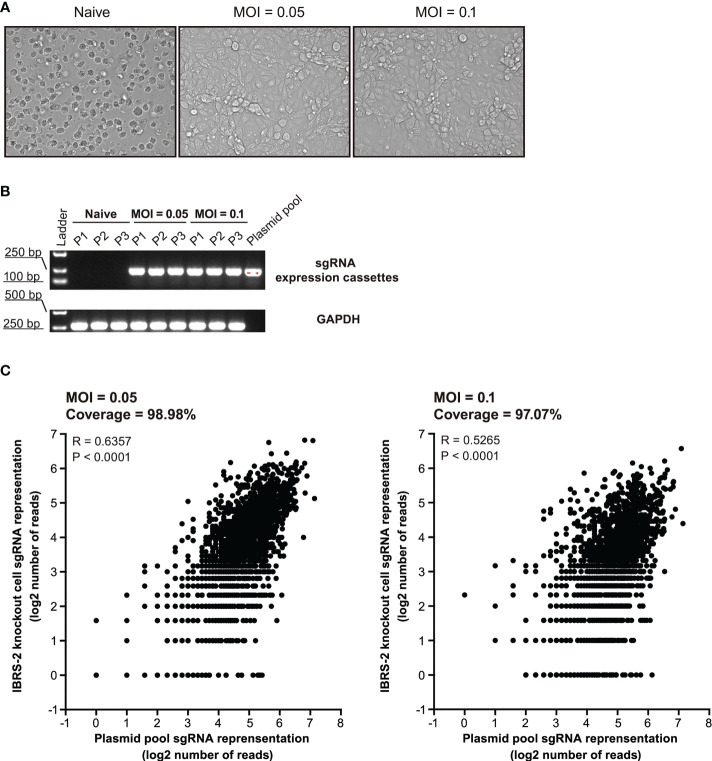
Construction and characterization of heterologous IBRS-2 knockout cell populations. **(A)** Cell morphology and growth characteristic of IBRS-2 knockout cell populations following three consecutive rounds of selective culture under the pressure of 5 μg/mL of puromycin. **(B)** Amplification of sgRNA expression cassettes using genomic DNA obtained from different passages of IBRS-2 knockout cell populations with plasmid pool as a positive control. GAPDH is used as an indicator of the input of genomic DNA. **(C)** Comparison of sgRNA representation between the plasmid pool to IBRS-2 knockout cell populations. Scatter plot of sgRNA representation between the plasmid pool to IBRS-2 knockout cell populations following three rounds of selective culture.

Substantial efforts have been aimed at screening which ISGs are antiviral and further uncovering their mechanisms-of-action. Loss-of-function screens based on CRISPR/Cas9 are gaining increasing popularity in identifying host factors that regulate virus infection. Based on previous RNA-Seq data, we constructed a CRISPR knockout library of 1908 unique sgRNAs targeting 5’ constitutive exons of 359 ISGs with an average coverage of 5 to 6 sgRNAs per gene. Following lentiviral transduction at varying MOI to attain no greater than 1 sgRNA per cell, and selective culture with puromycin (5 μg/mL) for 12 days, heterogeneous IBRS-2 knockout cell populations were harvested with high cell viability (**Figure 4A**). The integrity of ISG-targeting lentiCRISPR library in IBRS-2 knockout cell populations was confirmed by amplifying the sgRNA expression cassettes (**Figure 4B**). The scatter plot of sgRNA representation (log2 number of reads) signified that more than 98% sgRNAs from IBRS-2 knockout cell populations had representation in the plasmid pool, irrespective of the titer of lentivirus, further implying the reliability of IBRS-2 knockout cell populations (**Figure 4C**)

### Positive selection of gene knockouts that desensitize VSV-eGFP to type I IFN treatment

**Figure 5:**
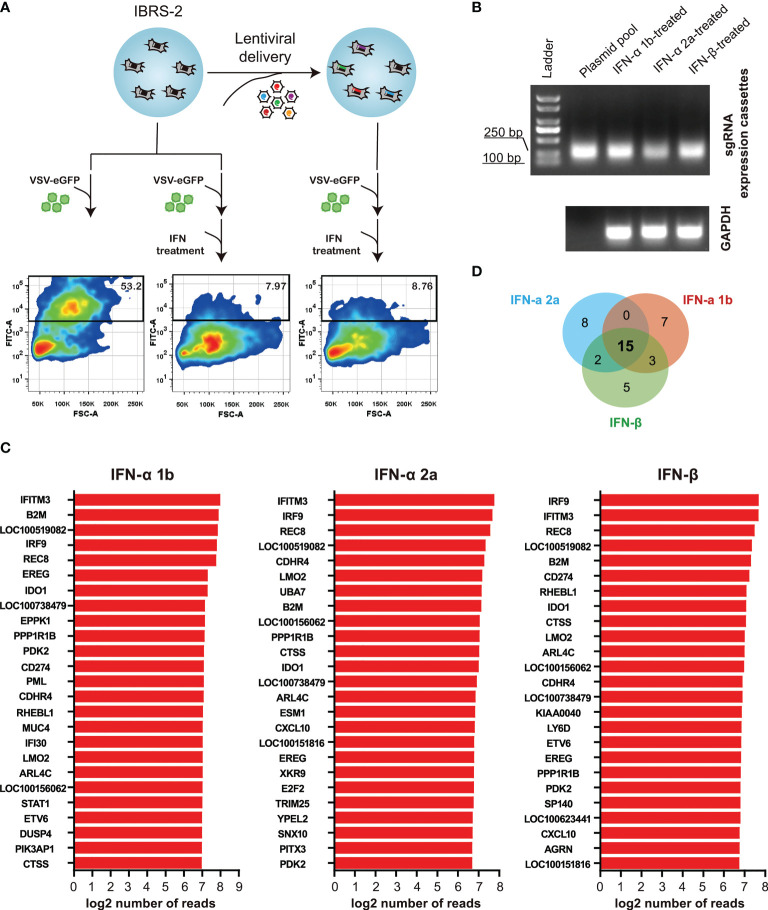
ISG-targeting CRISPR screen identifies a subset of genes as potential key effectors of the IFN response to VSV-eGFP replication. **(A)** Schematic of ISG-targeting lentiCRISPR screen to identify antiviral effectors mediating IFN-α 2a-induced antiviral response to VSV-eGFP. **(B)** Amplification of sgRNA expression cassettes in eGFP-positive IBRS-2 knockout cells. **(C)** Bar plots show the top 25 most enriched hits in the context of IFN-α 1b, IFN-α 2a and IFN-β. **(D)** Overlap between the top 25 most enriched ISGs in the context of IFN-α 1b, IFN-α 2a and IFN-β.

To enrich for sgRNAs that rendered cells permissive to VSV-eGFP replication despite treatment with a highly suppressive dose of type I IFNs, IBRS-2 knockout cell populations were infected with VSV-eGFP (0.1 MOI) followed by subsequent treatment with IFN-α 2a at a dose of 100 ng/mL. Following 36 h treatment, a cell population permissive to VSV-eGFP replication with high eGFP-signal intensity was enriched by FACS (**Figure 5A**). The same procedure was applied to IFN-α 1b and IFN-β at the same dose. Following amplifying the sgRNA expression cassettes in the enriched cells, sgRNA distribution was assessed by deep sequencing (**Figure 5B**). As expected, a diverse range of hits from the screening, with the demonstration of the top 25 most enriched ISGs, had the potential to reconstitute the IFN signaling pathway and served as effectors of the type I IFN antiviral response towards VSV-eGFP replication (**Figure 5C**). Comparing the top 25 hits revealed as much as 15 overlapping genes, as displayed in **Figure 5D** and **Table 1**. Noticeable is the fact that IRF9, a key component of the type I IFN pathway, was highly ranked in the present study. Broad-acting effectors, including LOC100519082, IFITM3 and REC8 previously described as inhibitors of VSV replication elsewhere, were also on the top of the list (19, 20).

**Table 1:** Details of fifteen overlapping highest-ranking hits obtained from IFN-α 1b, IFN-α 2a and IFN-β.

Gene_ID	product	Family
*IRF9*	Interferon regulatory factor 9	IRF
*IFITM3*	Interferon induced transmembrane protein 3	IFITM protein family
*REC8*	REC8 meiotic recombination protein	kleisin family of SMC protein partners
*LOC100519082*	Interferon induced transmembrane protein 1	IFITM protein family
*B2M*	Beta-2-microglobulin	
*PPP1R1B*	Protein phosphatase 1 regulatory inhibitor subunit 1B	
*LOC100156062*	Apolipoprotein L3	Apolipoprotein L gene family
*CDHR4*	Cadherin related family member 4	
*CTSS*	Cathepsin S	Peptidase C1 family
*LMO2*	LIM domain only 2	
*EREG*	Epiregulin	Epidermal growth factor (EGF) family
*IDO1*	Indoleamine 2, 3-dioxygenase 1	
*LOC100738479*	Tripartite motif-containing protein 34	
*ARL4C*	ADP ribosylation factor like GTPase 4C	ADP-ribosylation factor family of GTP-binding proteins
*PDK2*	Pyruvate dehydrogenase kinase 2	Pyruvate dehydrogenase kinase family

### Validation of the screening results by using overexpression assay

**Figure 6:**
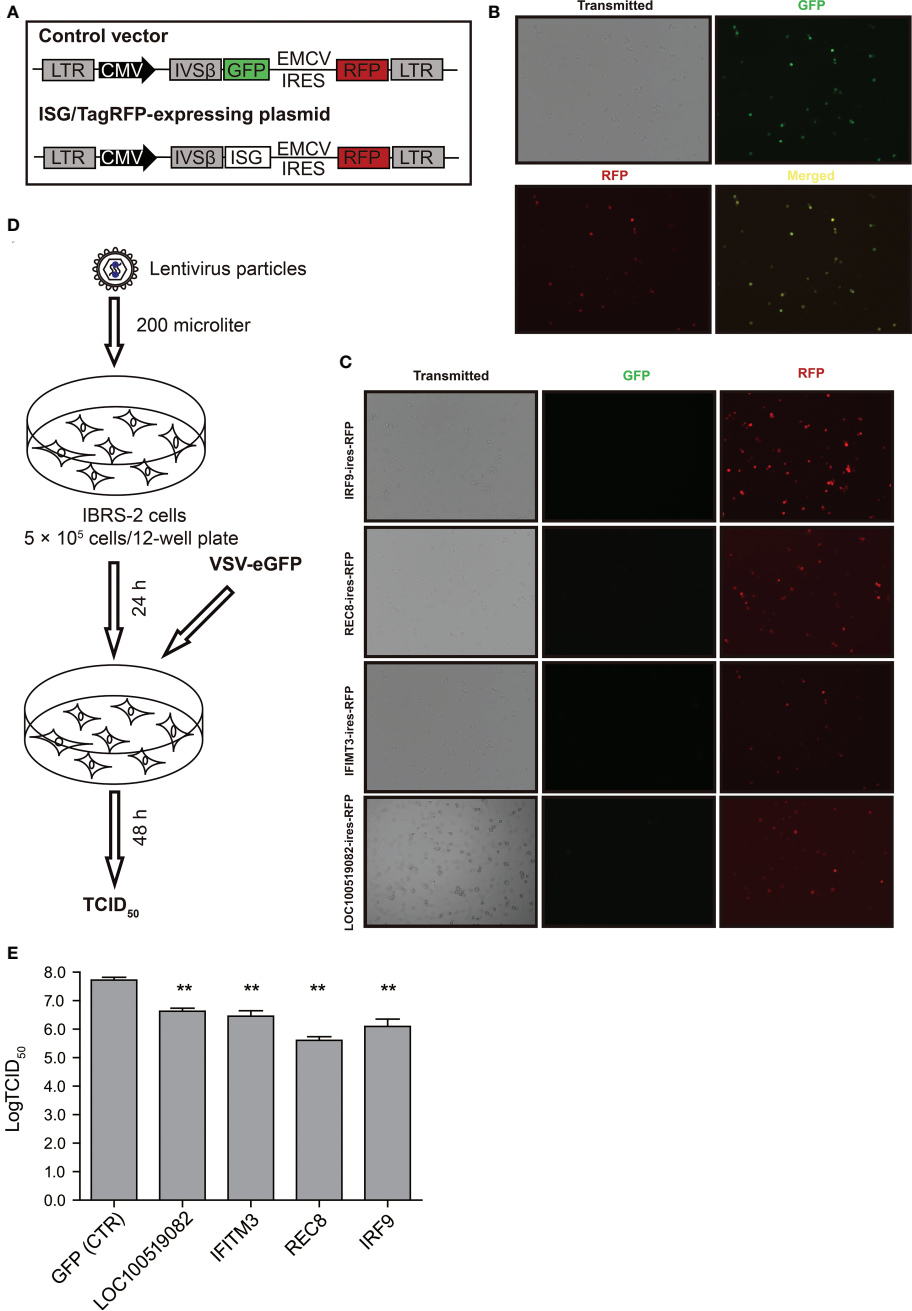
The effects of top four ISGs on VSV-eGFP replication. **(A)** Schematic representation of the gateway-compatible bicistronic lentiviral vectors used to stably overexpress ISGs. The viral backbone carries the ISG-IRES-TagRFP overexpression cassette under the CMV promoter. In parallel, control vector GFP-IRES-TagRFP was also designed. **(B)** Fluorescent micrographs of IBRS-2 cells in culture 24 h after transduction with the control vector GFP/TagRFP. **(C)** Fluorescent micrographs of IBRS-2 cells in culture 24 h after transduction with ISG/TagRFP vectors. **(D)** Schematic demonstration of workflow of transduction and virus infections. **(E)** The TCID_50_ titration of VSV-eGFP titers in the vector control and ISG/TagRFP overexpression IBRS-2 cells. The experiment was repeated three times with replicate each. **P < 0.01.

To validate the screening results, lentiviral overexpression of the top four candidate antiviral ISGs was performed to determine their effects on VSV-eGFP replication. The overexpression assay relied on a bicistronic lentiviral vector co-expressing an ISG and the red fluorescent protein TagRFP (**Figure 6A**). Of particular, the internal ribosomal entry site (IRES) from encephalomyocarditis virus (EMCV) was used in the bicistronic vector to initiate expression of an additional TagRFP, which functioned as a reporter signal indicative of successful overexpression of target gene. To explore the availability and translational efficacy of the bicistronic vector system, cells transduced with the control vector GFP/TagRFP were subjected to fluorescent microscopy. As displayed in **Figure 6B**, complete co-localization of GFP and RFP was observed in control cells, suggesting that adequate gene expression from the EMCV IRES element was achieved. We observed high intensities of RFP signals in IRF9/TagRFP, REC8/TagRFP, IFITM3/TagRFP and LOC100519082/TagRFP overexpression cells, indicative of high overexpression efficiency (**Figure 6C**). ISG/TagRFP-expressing cells were challenged with VSV-eGFP, and viral replication was titrated by TCID_50_ assay (**Figure 6D**). As a result, exogenous expressed IRF9 and REC8 conferred potent inhibition of VSV-eGFP replication, whereas LOC100519082 and IFITM3 only exerted moderate anti-VSV activity (**Figure 6E**).

## Discussion

The type I IFN system, resulting in the transcriptional elevation of hundreds of ISGs, constitutes the first line of defense against viral infections. The products of these ISGs exert distinct antiviral effector functions, a majority of which are still not well characterized. Meanwhile, each mammal owns a unique repertoire of ISGs, including genes conserved in all mammals and others specific to each species (21, 22). The recent efforts have been aimed at identifying the breadth of IFN-induced gene expression in a porcine cell line and constructing a validated gRNA library for CRISPR/Cas9 targeting of porcine 359 ISGs. Through extensive screening by using a model virus VSV-eGFP, several highest-ranking candidates have been enriched, including genes previously validated with anti-VSV activity and genes newly identified.

We prioritized IBRS-2 cell line above a couple of commercially available porcine cell lines in the present study. Firstly, IBRS-2 cells are compromised in activation of RIG-I-like signaling pathway during virus infection. Consequently, virus stimulation is insufficient in strong and protracted induction of genes related to IFN, inflammatory, and innate immune response in IBRS-2 cells (12). Secondly, IBRS-2 cells possess an intact type I IFN pathway. Addition of exogenous type I IFNs could activate the downstream JAK-STAT pathway and result in transcriptions of ISGs (23). According to the principle of CRISPR screening strategies in **Figure 5A**, cells were first infected with the virus followed by IFN treatment. Thus, an aberrant RLR pathway in IBRS-2 cells could exclude the interference of endogenous IFN, inflammatory, and concurrent innate immune responses with exogenous IFN treatment, guaranteeing the accuracy of CRISPR screening to the greatest extent. Meanwhile, VSV-eGFP was applied for CRISPR screening. The VSV is a negative-sense single-stranded RNA virus. It is named as per the resultant classical vesicular lesions in affected natural hosts, such as horses, cattle, and pigs (24, 25). It is a widely used model system for virus-IFN interaction, likely due to its high sensitivity to IFN treatment (26). Although the present investigation was limited to VSV-eGFP, this library can be applied to a much wider range of viruses, such as Senecavirus A (SVA) and foot-and-mouth disease virus (FMDV) (11, 27). Infection with those viruses caused cytopathic effect (CPE) in IBRS-2 cells, yielding high titers of viral suspension.

One limitation of the present research is devoid of validation of all the top 15 hits against VSV-eGFP replication by pharmacological and genetic approaches. However, among the highest-ranking hits with generic biological effects, some were previously noted to have anti-VSV activity: (I) IRF9, a key component of the JAK-STAT pathway, functioned as a broadly acting effector against a list of viruses (28, 29). Besides, a loss-of-function screen using a small interfering RNA (siRNA) library identified IRF9 as the most significantly enriched hit responsible for the activity of IFN-α against VSV, Indiana serotype (VSV_IND_) (30). (II) IFITM1/IFITM3 restricted VSV replication potentially through toughening the host membrane, thus preventing viral membrane fusion (19); (III) REC8 promoted the innate immune response by targeting STING and MAVS, thus constraining VSV replication (20). Noticeable is the fact that those four hits are on the top of list. Confirmation of top 4 hits *via* individual gene overexpression demonstrated the same findings, convincingly indicative of the feasibility and reliability of this knockout library. In future work, the antiviral activities and exact mechanism-of-action of novel hits need to be further characterized.

ISG products constitute a complex web of host defenses and take on a number of diverse roles. The IBRS-2 knockout cell populations contained 98% sgRNAs compared with the plasmid library, perhaps due to the fact that some sgRNAs may target ISGs essential for cell survival. Besides, ISG products exert their antiviral activity through multiple mechanisms. Many of the ISG products directly disrupt a particular step in the infection/replication cycle. For example, it is well noted that IFITM3 inhibited viral entry (31, 32). In addition, multiple ISGs (such as IRF1, IRF9 and REC8) likely conferred inhibition of viral replication by amplifying host antiviral state by stimulating IFN expression or interferon-stimulated response element (ISRE)-driven transcription (33). Conversely, several ISGs functioned as negative regulators of IFN signaling pathways and conferred cultured cells an IFN-desensitized state shortly after IFN exposure. SOCS and USP18 were two well-known ISGs that negatively regulate IFN signaling by inhibiting the JAK-STAT signaling pathway (34, 35). However, those ISGs were not highly enriched in the present screening. One of the reasons is that the intrinsic expression of ISGs in IBRS-2 is relatively low. Following IFN stimulation the ISG expression level increased to a level at which the cells are easy to bear.

In summary, this study presents a versatile CRISPR/Cas9 knockout library targeting 359 selected porcine ISGs with predesigned and validated sgRNAs and complete protocols for screenings. The results have expanded the ISG library in pig species and provide additional evidence that CRISPR/Cas9 system is suitable for screening novel ISGs. Furthermore, the antiviral activities of the new ISGs and their biological functions against VSV await further investigation.

## Data availability statement

The data presented in the study are deposited in the Sequence Read Archive (SRA) repository, accession number PRJNA899479.

## Author contributions

Conceptualization, WD, TL, and HZ. Methodology, WD, TL and FX. Software, YW and FY. Validation, WD and TL. Formal analysis, WD and TL. Data curation, WD and TL. Writing—original draft preparation, WD and TL. Writing—review and editing, WD, TL, and HZ. Visualization, FX and YW. Supervision, HZ. Project administration, WD and HZ. Funding acquisition, WD and HZ. All authors contributed to the article and approved the submitted version.

## Funding

This work was funded by grants from the National Key R&D Program of China (2021YFD1800300), the State Key Laboratory of Veterinary Biotechnology (SKLVEB2021DBCG01), the Gansu Provincial Major Project for Science and Technology Development (19ZD2NA001 and 21ZD3NA001), the Central Public-interest Scientific Institution Basal Research Fund (to WD and FY), the Chinese Academy of Agricultural Science and Technology Innovation Project (CAAS-ASTIP-2022-LVRI), the Earmarked Fund for CARS-35, the Open Competition Program of Top Ten Critical Priorities of Agricultural Science and Technology Innovation for the 14th Five-Year Plan of Guangdong Province (2022SDZG02) and the Natural Science Foundation of Gansu Province (22JR5RA996).

## Conflict of interest

The authors declare that the research was conducted in the absence of any commercial or financial relationships that could be construed as a potential conflict of interest.

## Publisher’s note

All claims expressed in this article are solely those of the authors and do not necessarily represent those of their affiliated organizations, or those of the publisher, the editors and the reviewers. Any product that may be evaluated in this article, or claim that may be made by its manufacturer, is not guaranteed or endorsed by the publisher.
