# Supplementary figures and images for: Establishment of a CRISPR/Cas9 knockout library for screening type I interferon-inducible antiviral effectors in pig cells
Source: Front Immunol. 2022 Nov 24;13:1016545. doi: 10.3389/fimmu.2022.1016545 (PMC9732717; doi:10.3389/fimmu.2022.1016545)

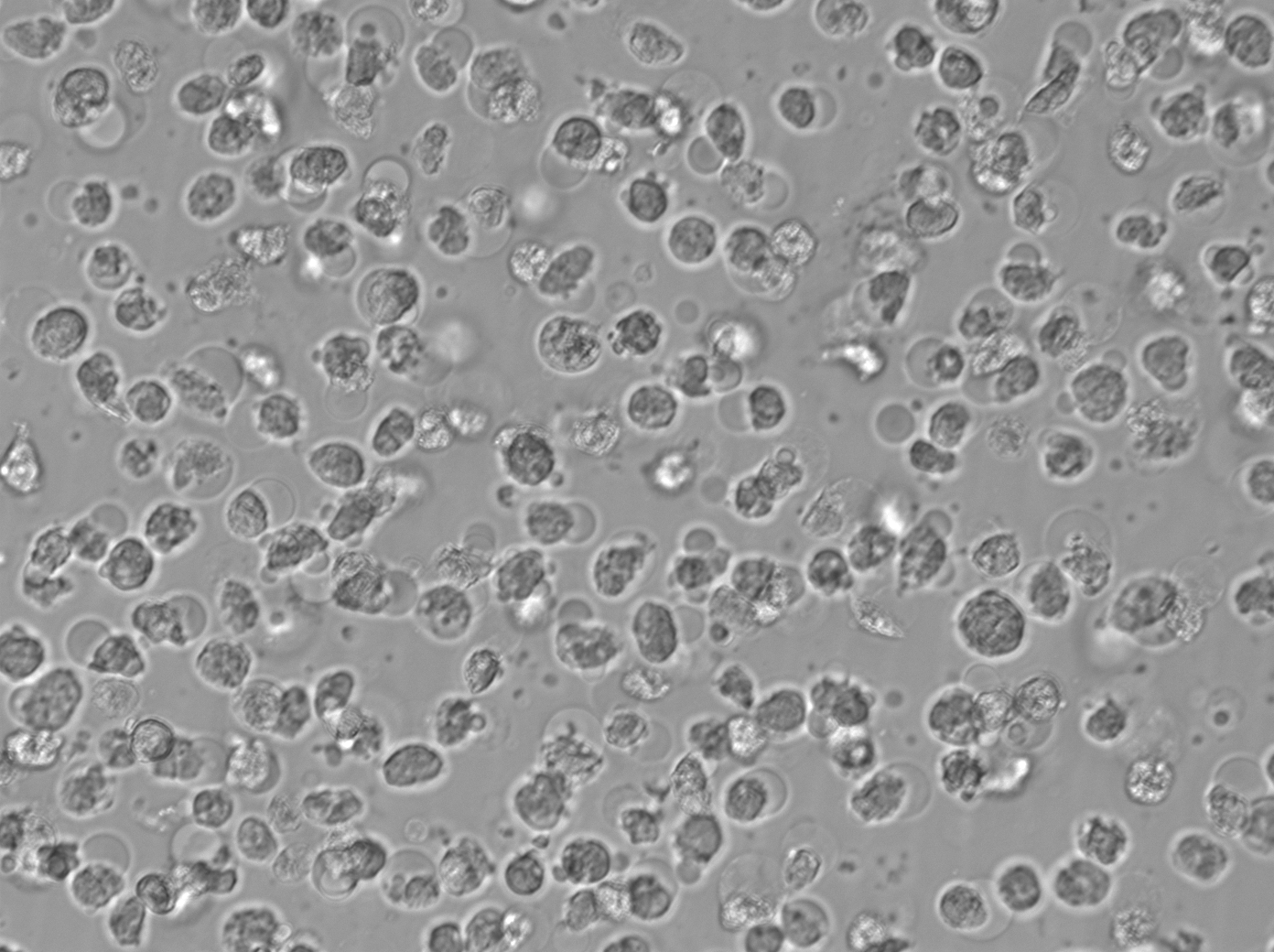

Supplement: Supplementary file 1 [file Image_1.tif]

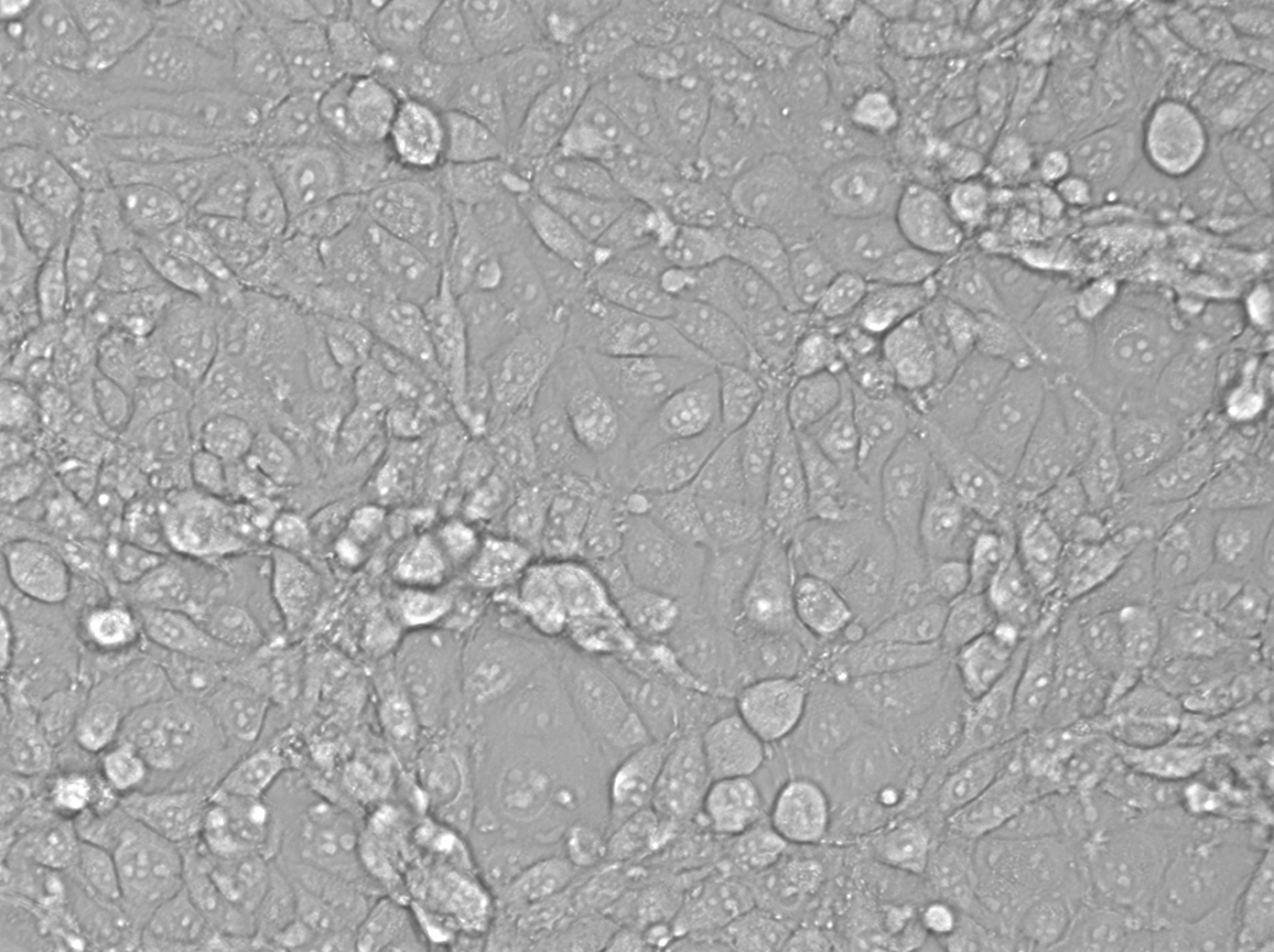

Supplement: Supplementary file 2 [file Image_2.tif]

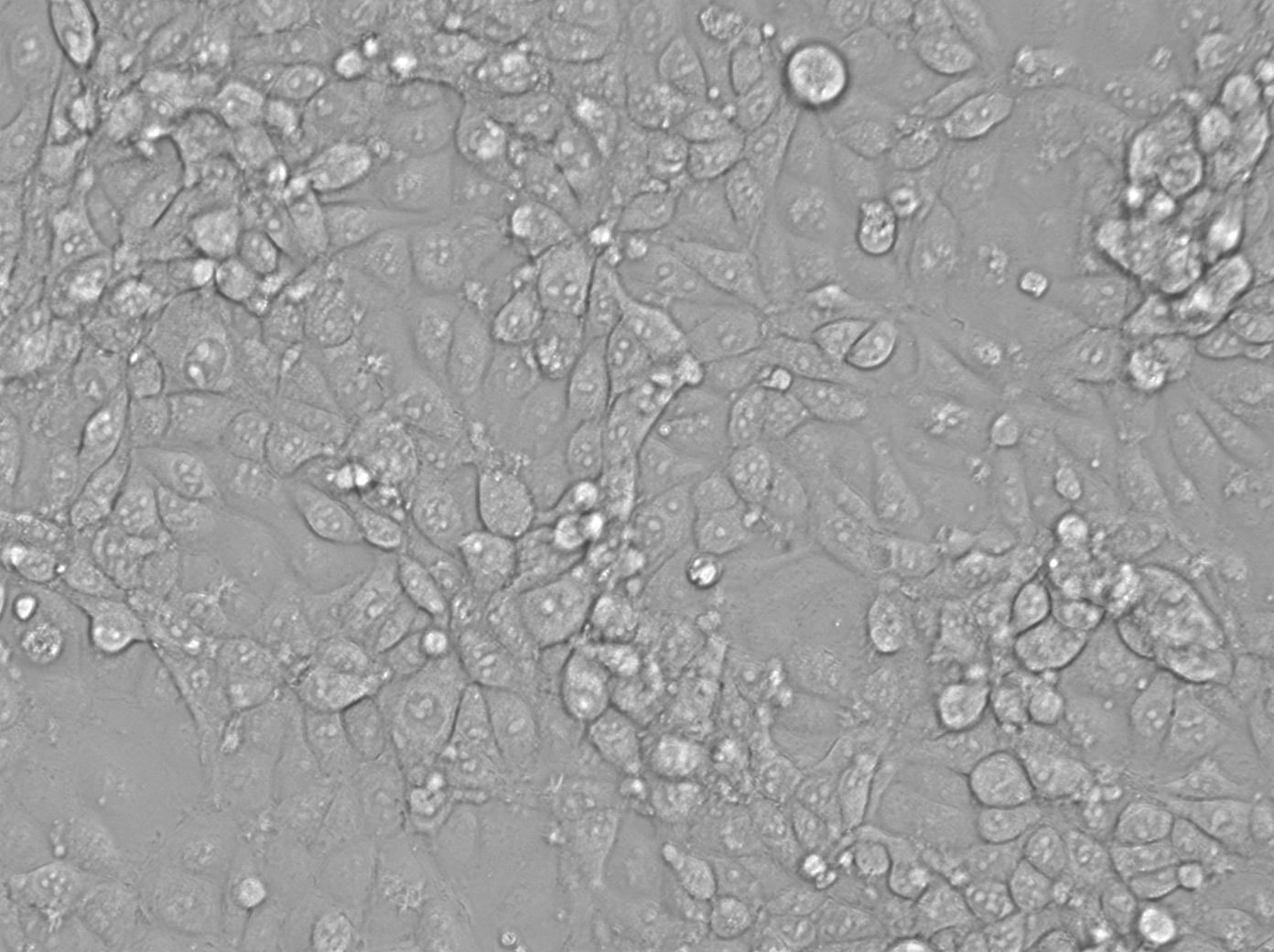

Supplement: Supplementary file 3 [file Image_3.tif]

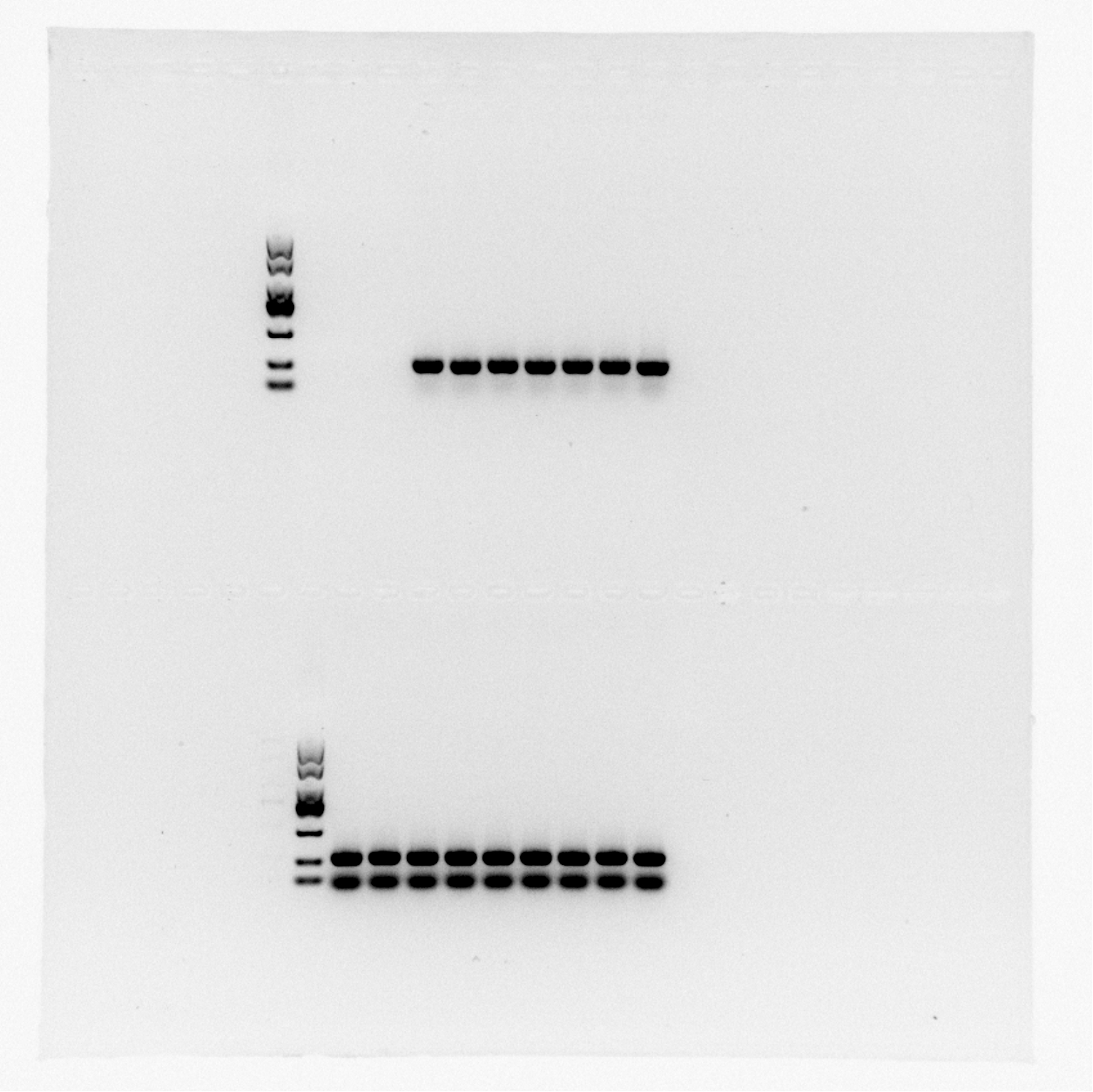

Supplement: Supplementary file 4 [file Image_4.jpeg]

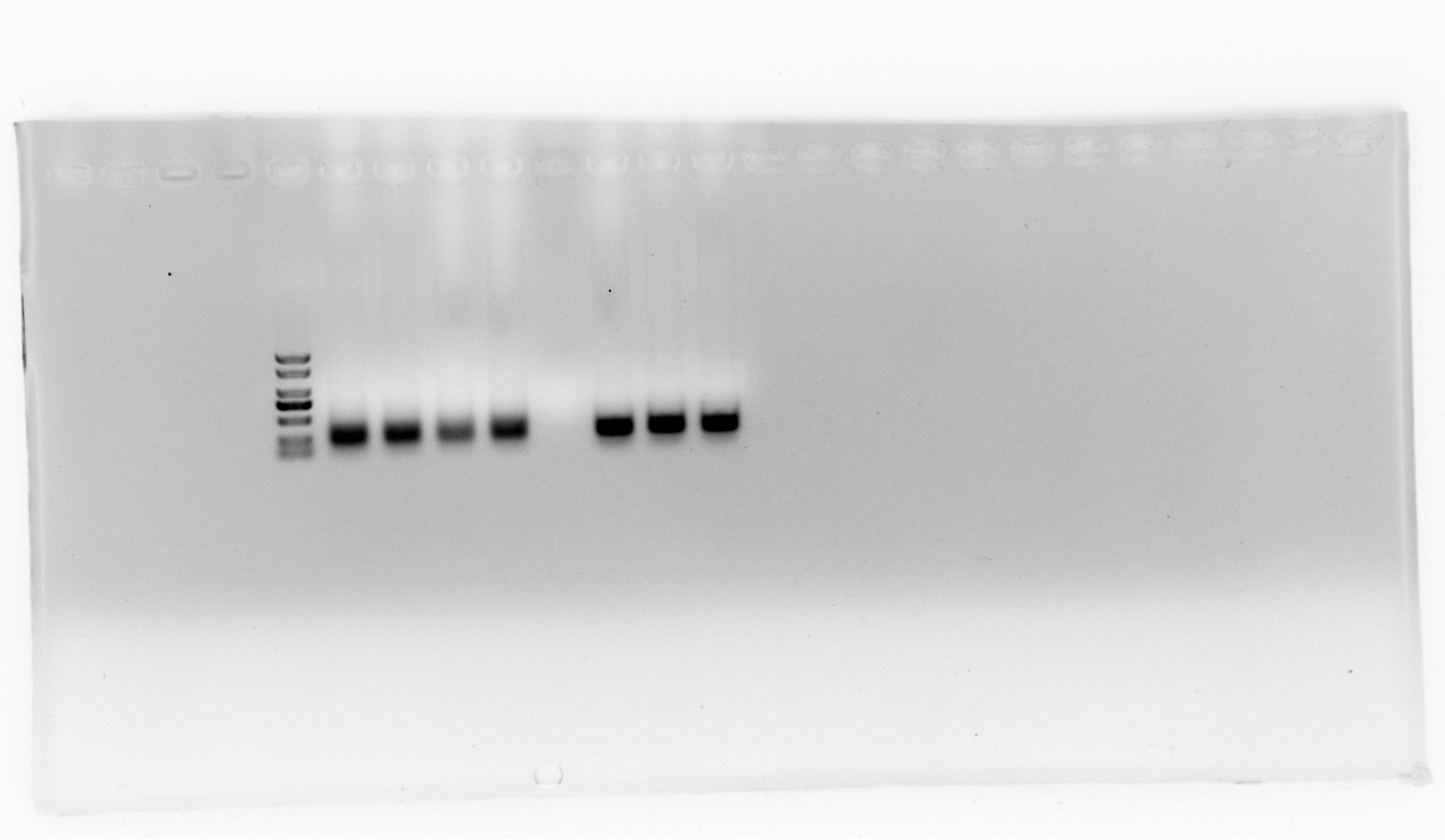

Supplement: Supplementary file 5 [file Image_5.jpeg]
