# Supplementary material for: Establishment of a CRISPR/Cas9 knockout library for screening type I interferon-inducible antiviral effectors in pig cells
Source: Front Immunol. 2022 Nov 24;13:1016545. doi: 10.3389/fimmu.2022.1016545 (PMC9732717; doi:10.3389/fimmu.2022.1016545)
Supplement: Supplementary file 11 [file DataSheet_1.docx]

**Supplementary Data**

**Establishment of a CRISPR/Cas9 knockout library for screening type I interferon-inducible antiviral effectors in pig cells**

Wen Dang^1†^, Tao Li^1†^, Fan Xu^1^, Yannan Wang^2^, Fan Yang^1^, Haixue Zheng^1*^

*^1^State Key Laboratory of Veterinary Etiological Biology, College of Veterinary Medicine, Lanzhou University, Lanzhou Veterinary Research Institute, Chinese Academy of Agricultural Sciences, Lanzhou 730046, China*

*^2^Lanzhou University Second Hospital, the Department of Radiology.*

* Corresponding authors.

[haixuezheng@163.com](mailto:haixuezheng@163.com) (H. Zheng)

^†^ Wen Dang and Tao Li contributed equally to this work.

**Supplementary Table 1 Details of selected ISGs in Fig. 3.**

| Gene_id | Dbxref | Roduct |
| --- | --- | --- |
| ADAR | GeneID: 100523784,  Genbank: XM_021089756.1 | adenosine deaminase, RNA specific |
| ADM | GeneID:397195,  Genbank: NM_214107.1 | adrenomedullin |
| AGRN | GeneID:100621139,  Genbank: XM_021095177.1 | agrin |
| AKAP7 | GeneID:100523651,  Genbank: XM_021088003.1 | A-kinase anchoring protein 7 |
| ALPL | GeneID:100170147,  Genbank: XM_021097682.1 | alkaline phosphatase, liver/bone/kidney |
| AMIGO2 | GeneID:100152349,  Genbank: XM_005664161.3 | adhesion molecule with Ig like domain 2 |
| APOBEC3B | GeneID:100037939,  Genbank: NM_001097446.1 | apolipoprotein B mRNA editing enzyme catalytic subunit 3B |
| ARHGAP8 | GeneID:100533202,  Genbank: XM_021091086.1 | Rho GTPase activating protein 8 |
| ARID5A | GeneID:100524615,  Genbank: XM_021087092.1 | AT-rich interaction domain 5A |
| ASB9 | GeneID:100158057,  Genbank: XM_013985902.2 | ankyrin repeat and SOCS box containing 9 |
| ASPHD2 | GeneID:100156894,  Genbank: XM_001926554.5 | aspartate beta-hydroxylase domain containing 2 |
| ATAD1 | GeneID:100157471,  Genbank: XM_005671257.3 | ATPase family, AAA domain containing 1 |
| ATF3 | GeneID:100738612,  Genbank: XM_021063853.1 | activating transcription factor 3 |
| ATP10D | GeneID:100520087,  Genbank: XM_021100567.1 | ATPase phospholipid transporting 10D (putative) |
| B2M | GeneID:110255236,  Genbank: XM_021096362.1 | beta-2-microglobulin |
| BATF2 | GeneID:100515875,  Genbank: XM_013994125.2 | basic leucine zipper ATF-like transcription factor 2 |
| BCL2L14 | GeneID:100514901,  Genbank: NM_001315668.1 | BCL2 like 14 |
| BIRC3 | GeneID:100622590,  Genbank: XM_021062707.1 | baculoviral IAP repeat containing 3 |
| BRCA1 | GeneID:100049662,  Genbank: XM_021066931.1 | BRCA1, DNA repair associated |
| BST2 | GeneID:100302088,  Genbank: NM_001161755.1 | bone marrow stromal cell antigen 2 |
| BTC | GeneID:100505411,  Genbank: XM_021101433.1 | betacellulin |
| C12H17orf100 | GeneID:100521293,  Genbank: XM_021067715.1 | chromosome 12 C17orf100 homolog |
| C1R | GeneID:445464,  Genbank: XM_021092408.1 | complement C1r |
| C1S | GeneID:397274,  Genbank: XM_021091133.1 | complement C1s |
| C2 | GeneID:448981,  Genbank: NM_001101815.1 | complement C2 |
| C2CD3 | GeneID:100514911,  Genbank: XM_021062487.1 | C2 calcium dependent domain containing 3 |
| C2H19orf66 | GeneID:100512458,  Genbank: NM_001244321.1 | chromosome 2 C19orf66 homolog |
| C3 | GeneID:397072,  Genbank: XM_021080819.1 | complement C3 |
| C8H4orf19 | GeneID:100738623,  Genbank: XM_021101097.1 | chromosome 8 C4orf19 homolog |
| CACNG7 | GeneID:100519605,  Genbank: XM_003127406.5 | calcium voltage-gated channel auxiliary subunit gamma 7 |
| CADPS2 | GeneID:100525671,  Genbank: XM_021078970.1 | calcium dependent secretion activator 2 |
| CAMK2D | GeneID:397674,  Genbank: NM_214381.1 | calcium/calmodulin dependent protein kinase II delta |
| CASP10 | GeneID:100154896,  Genbank: XM_021074526.1 | caspase 10 |
| CASP7 | GeneID:100156777,  Genbank: XM_021073322.1 | caspase 7 |
| CASZ1 | GeneID:100510911,  Genbank: XM_021095326.1 | castor zinc finger 1 |
| CCL2 | GeneID:397422,  Genbank: NM_214214.1 | chemokine (C-C motif) ligand 2 |
| CCL4 | GeneID:396668,  Genbank: NM_213779.1 | C-C motif chemokine ligand 4 |
| CCL5 | GeneID:396613,  Genbank: NM_001129946.1 | C-C motif chemokine ligand 5 |
| CD274 | GeneID:574058,  Genbank: XM_021083736.1 | CD274 molecule |
| CD86 | GeneID:397441,  Genbank: XM_005654045.3 | CD86 molecule |
| CDHR4 | GeneID:100513757,  Genbank: XM_021068791.1 | cadherin related family member 4 |
| CFI | GeneID:100516921,  Genbank: XM_013989347.2 | complement factor I |
| CHMP7 | GeneID:110256869,  Genbank: XM_021074113.1 | charged multivesicular body protein 7 |
| CIITA | GeneID:106509697,  Genbank: XM_021085809.1 | class II major histocompatibility complex transactivator |
| CLIC5 | GeneID:100154589,  Genbank: NM_001198923.2 | chloride intracellular channel 5 |
| CLMP | GeneID:100512427,  Genbank: XM_021063025.1 | CXADR like membrane protein |
| CMTR1 | GeneID:100154061,  Genbank: XM_001924210.5 | cap methyltransferase 1 |
| CNP | GeneID:100511587,  Genbank: XM_003131420.5 | 2',3'-cyclic nucleotide 3' phosphodiesterase |
| COBLL1 | GeneID:100737004,  Genbank: XM_021075418.1 | cordon-bleu WH2 repeat protein like 1 |
| CPNE8 | GeneID:100519777,  Genbank: XM_021092523.1 | copine 8 |
| CSF1 | GeneID:100513084,  Genbank: XM_021088654.1 | colony stimulating factor 1 |
| CTSS | GeneID:100153090,  Genbank: XM_021089893.1 | cathepsin S |
| CXCL10 | GeneID:494019,  Genbank: NM_001008691.1 | C-X-C motif chemokine ligand 10 |
| CXCL11 | GeneID:100169744,  Genbank: XM_005666774.3 | C-X-C motif chemokine ligand 11 |
| CXCL2 | GeneID:414904,  Genbank: NM_001001861.2 | chemokine (C-X-C motif) ligand 2 |
| CXCL8 | GeneID:396880,  Genbank: NM_213867.1 | C-X-C motif chemokine ligand 8 |
| CYP4V2 | GeneID:100113469,  Genbank: XM_021075586.1 | cytochrome P450, family 4, subfamily v, polypeptide 2 |
| DAPP1 | GeneID:100525936,  Genbank: XM_013979010.2 | dual adaptor of phosphotyrosine and 3-phosphoinositides 1 |
| DAXX | GeneID:100524813,  Genbank: XM_021098591.1 | death domain associated protein |
| DDX58 | GeneID:396723,  Genbank: XM_021064058.1 | DExD/H-box helicase 58 |
| DDX60 | GeneID:100158037,  Genbank: XM_021072214.1 | DExD/H-box helicase 60 |
| DHX58 | GeneID:100524520,  Genbank: XM_005668815.3 | DExH-box helicase 58 |
| DTX3L | GeneID:100520459,  Genbank: XM_021070271.1 | deltex E3 ubiquitin ligase 3L |
| E2F2 | GeneID:110261113,  Genbank: XM_021095527.1 | E2F transcription factor 2 |
| EDNRA | GeneID:397457,  Genbank: XM_021100476.1 | endothelin receptor type A |
| EHD4 | GeneID:100738234,  Genbank: XM_021097351.1 | EH domain containing 4 |
| EHF | GeneID:100526094,  Genbank: XM_013994435.2 | ETS homologous factor |
| EIF2AK2 | GeneID:397588,  Genbank: XM_021085862.1 | eukaryotic translation initiation factor 2 alpha kinase 2 |
| ENPP4 | GeneID:100738210,  Genbank: XM_001924940.6 | ectonucleotide pyrophosphatase/phosphodiesterase 4 (putative) |
| ENTPD4 | GeneID:100155254,  Genbank: XM_021072268.1 | ectonucleoside triphosphate diphosphohydrolase 4 |
| EPCAM | GeneID:403163,  Genbank: NM_214419.1 | epithelial cell adhesion molecule |
| EPPK1 | GeneID:110260406,  Genbank: XM_021090394.1 | epiplakin 1 |
| EPSTI1 | GeneID:100625050,  Genbank: XM_003482896.4 | epithelial stromal interaction 1 |
| ERAP1 | GeneID:100523953,  Genbank: XM_021085396.1 | endoplasmic reticulum aminopeptidase 1 |
| ERAP2 | GeneID:100525036,  Genbank: XM_021085395.1 | endoplasmic reticulum aminopeptidase 2 |
| ESM1 | GeneID:100739671,  Genbank: XM_013984771.2 | endothelial cell specific molecule 1 |
| ETV6 | GeneID:100157726,  Genbank: XM_005652587.3 | ETS variant 6 |
| ETV7 | GeneID:100156210,  Genbank: XM_013977848.2 | ETS variant 7 |
| FAM46A | GeneID:100156873,  Genbank: XM_013992805.2 | family with sequence similarity 46 member A |
| FBXO39 | GeneID:106504168,  Genbank: XM_013981237.2 | F-box protein 39 |
| FOXS1 | GeneID:102167710,  Genbank: XM_021077448.1 | forkhead box S1 |
| FRY | GeneID:100155799,  Genbank: XM_021065177.1 | FRY microtubule binding protein |
| FUNDC1 | GeneID:100518350,  Genbank: XM_003135038.4 | FUN14 domain containing 1 |
| GBP1 | GeneID:100151938,  Genbank: XM_021088503.1 | guanylate binding protein 1, interferon-inducible |
| GBP2 | GeneID:100153137,  Genbank: NM_001128474.1 | guanylate binding protein 2, interferon-inducible |
| GGN | GeneID:100515664,  Genbank: XM_005664585.3 | gametogenetin |
| GJD3 | GeneID:100522209,  Genbank: XM_013980984.2 | gap junction protein delta 3 |
| GM2A | GeneID:100515283,  Genbank: XM_013984919.2 | GM2 ganglioside activator |
| GMPR | GeneID:100154573,  Genbank: XM_001929120.7 | guanosine monophosphate reductase |
| GNG11 | GeneID:100522202,  Genbank: XM_005667613.3 | G protein subunit gamma 11 |
| GPR155 | GeneID:100511231,  Genbank: XM_013990589.2 | G protein-coupled receptor 155 |
| GUCA1A | GeneID:100739200,  Genbank: XM_021098697.1 | guanylate cyclase activator 1A |
| GVIN1 | GeneID:100512797,  Genbank: XM_003482521.4 | GTPase, very large interferon inducible 1 |
| HERC5 | GeneID:100518083,  Genbank: XM_021100766.1 | HECT and RLD domain containing E3 ubiquitin protein ligase 5 |
| HERC6 | GeneID:100626657,  Genbank: XM_021100765.1 | HECT and RLD domain containing E3 ubiquitin protein ligase family member 6 |
| HES4 | GeneID:100739264,  Genbank: XM_003481929.4 | hes family bHLH transcription factor 4 |
| HHLA2 | GeneID:102166962,  Genbank: XM_005670246.3 | HERV-H LTR-associating 2 |
| HS3ST1 | GeneID:100628223,  Genbank: XM_021101019.1 | heparan sulfate-glucosamine 3-sulfotransferase 1 |
| IDO1 | GeneID:100519877,  Genbank: NM_001246240.1 | indoleamine 2,3-dioxygenase 1 |
| IFI30 | GeneID:100174943,  Genbank: NM_001131046.1 | IFI30, lysosomal thiol reductase |
| IFI35 | GeneID:100624758,  Genbank: XM_003358024.3 | interferon induced protein 35 |
| IFI44 | GeneID:100525523,  Genbank: NM_001246205.1 | interferon induced protein 44 |
| IFI44L | GeneID:100511267,  Genbank: XM_003127919.4 | interferon-induced protein 44-like |
| IFI6 | GeneID:110261124,  Genbank: XM_021095660.1 | interferon alpha inducible protein 6 |
| IFIH1 | GeneID:100101927,  Genbank: XM_005671881.3 | interferon induced with helicase C domain 1 |
| IFIT1 | GeneID:100153038,  Genbank: NM_001244363.1 | interferon-induced protein with tetratricopeptide repeats 1 |
| IFIT2 | GeneID:100155467,  Genbank: XM_005671264.3 | interferon induced protein with tetratricopeptide repeats 2 |
| IFIT3 | GeneID:100154248,  Genbank: XM_021071929.1 | interferon induced protein with tetratricopeptide repeats 3 |
| IFITM1 | GeneID:100127358,  Genbank: XM_003124230.2 | interferon-induced transmembrane protein 1 |
| IFITM3 | GeneID:100518544,  Genbank: NM_001201382.1 | interferon induced transmembrane protein 3 |
| IL12RB1 | GeneID:100271900,  Genbank: XM_013987467.2 | interleukin 12 receptor subunit beta 1 |
| IL15RA | GeneID:733692,  Genbank: XM_021065032.1 | interleukin 15 receptor subunit alpha |
| IL6 | GeneID:399500,  Genbank: NM_214399.1 | interleukin 6 |
| IL7 | GeneID:397253,  Genbank: XM_013996658.2 | interleukin 7 |
| IRF1 | GeneID:396611,  Genbank: XM_021080244.1 | interferon regulatory factor 1 |
| IRF2 | GeneID:100621620,  Genbank: XM_021075622.1 | interferon regulatory factor 2 |
| IRF7 | GeneID:100037289,  Genbank: XM_021076250.1 | interferon regulatory factor 7 |
| IRF9 | GeneID:780415,  Genbank: NM_001078670.1 | interferon regulatory factor 9 |
| IRS1 | GeneID:100512686,  Genbank: NM_001244489.1 | insulin receptor substrate 1 |
| ISG12(A) | GeneID:100153902,  Genbank: NM_001198921.1 | putative ISG12(a) protein |
| ISG15 | GeneID:100145895,  Genbank: NM_001128469.2 | ISG15 ubiquitin-like modifier |
| ISG20 | GeneID:448855,  Genbank: XM_021062570.1 | interferon stimulated exonuclease gene 20 |
| KHNYN | GeneID:100158093,  Genbank: XM_021099195.1 | KH and NYN domain containing |
| KIAA0040 | GeneID:100519616,  Genbank: XM_013989639.2 | KIAA0040 ortholog |
| KLF8 | GeneID:100154631,  Genbank: XM_021079404.1 | Kruppel like factor 8 |
| LGALS3BP | GeneID:110255948,  Genbank: XM_021066516.1 | galectin 3 binding protein |
| LGALS8 | GeneID:100156193,  Genbank: XM_021071989.1 | galectin 8 |
| LGALS9 | GeneID:396972,  Genbank: XM_021066200.1 | galectin 9 |
| LMO2 | GeneID:100512825,  Genbank: XM_005661026.2 | LIM domain only 2 |
| LOC100151816 | GeneID:100151816,  Genbank: NM_001315572.1 | interferon-induced protein with tetratricopeptide repeats 5 |
| LOC100155195 | GeneID:100155195,  Genbank: XM_021090310.1 | guanylate-binding protein 7 |
| LOC100156062 | GeneID:100156062,  Genbank: XM_021091541.1 | apolipoprotein L3 |
| LOC100517129 | GeneID:100517129,  Genbank: XM_021075662.1 | nuclear body protein SP140-like protein |
| LOC100519082 | GeneID:100519082,  Genbank: XM_003124235.5 | interferon-induced transmembrane protein 1 |
| LOC100519098 | GeneID:100519098,  Genbank: XM_021102382.1 | sterile alpha motif domain-containing protein 9 |
| LOC100520273 | GeneID:100520273,  Genbank: XM_021069823.1 | poly [ADP-ribose] polymerase 15 |
| LOC100522887 | GeneID:100522887,  Genbank: XM_003129812.6 | caspase-13 |
| LOC100523310 | GeneID:100523310,  Genbank: XM_005663706.3 | guanylate-binding protein 6 |
| LOC100523492 | GeneID:100523492,  Genbank: XM_013997408.2 | guanylate-binding protein 5 |
| LOC100523668 | GeneID:100523668,  Genbank: XM_021090315.1 | guanylate-binding protein 2-like |
| LOC100620827 | GeneID:100620827,  Genbank: XM_021091411.1 | uncharacterized LOC100620827 |
| LOC100623441 | GeneID:100623441,  Genbank: XM_013995888.1 | sulfotransferase 1C4 |
| LOC100626247 | GeneID:100626247,  Genbank: XM_003354408.4 | interferon-induced transmembrane protein 1-like |
| LOC100626318 | GeneID:100626318,  Genbank: XM_005664804.3 | cationic amino acid transporter 3-like |
| LOC100626407 | GeneID:100626407,  Genbank: XM_013998732.2 | cationic amino acid transporter 3 |
| LOC100738479 | GeneID:100738479,  Genbank: XM_005667079.3 | tripartite motif-containing protein 34 |
| LOC102161784 | GeneID:102161784,  Genbank: XM_005663708.3 | guanylate-binding protein 6-like |
| LOC106510284 | GeneID:106510284,  Genbank: XM_021091543.1 | apolipoprotein L3-like |
| LOC110255360 | GeneID:110255360,  Genbank: XM_021062326.1 | interferon-induced very large GTPase 1-like |
| LOC110255361 | GeneID:110255361,  Genbank: XM_021062330.1 | interferon-induced very large GTPase 1-like |
| LOC110256626 | GeneID:110256626,  Genbank: XM_021072532.1 | proline dehydrogenase 1, mitochondrial |
| LOC110260835 | GeneID:110260835,  Genbank: XM_021093099.1 | uncharacterized LOC110260835 |
| LOC110261384 | GeneID:110261384,  Genbank: XM_021097585.1 | translation initiation factor IF-2-like |
| LOC733579 | GeneID:733579,  Genbank: NM_001044532.1 | tripartite motif protein TRIM5 |
| LOC780435 | GeneID:780435,  Genbank: XM_021088755.1 | glutathione S-transferase mu 2 |
| LRRN4CL | GeneID:100620756,  Genbank: XM_003353820.4 | LRRN4 C-terminal like |
| MARCKSL1 | GeneID:100628001,  Genbank: XM_003356281.4 | MARCKS like 1 |
| MAST3 | GeneID:100736673,  Genbank: XM_021083495.1 | microtubule associated serine/threonine kinase 3 |
| MFSD12 | GeneID:100519240,  Genbank: XM_021084115.1 | major facilitator superfamily domain containing 12 |
| MMD2 | GeneID:100512096,  Genbank: XM_003124274.5 | monocyte to macrophage differentiation associated 2 |
| MORC3 | GeneID:100513379,  Genbank: XM_003132764.4 | MORC family CW-type zinc finger 3 |
| MOV10 | GeneID:100521086,  Genbank: XM_005655442.3 | Mov10 RISC complex RNA helicase |
| MUC4 | GeneID:100157344,  Genbank: XM_021068272.1 | mucin 4, cell surface associated |
| MX1 | GeneID:397128,  Genbank: NM_214061.2 | MX dynamin like GTPase 1 |
| MX2 | GeneID:396893,  Genbank: NM_001097416.1 | myxovirus (influenza virus) resistance 2 (mouse) |
| MXD1 | GeneID:100625349,  Genbank: XM_003354782.5 | MAX dimerization protein 1 |
| MYO1G | GeneID:100512998,  Genbank: XM_021079179.1 | myosin IG |
| N4BP1 | GeneID:100520431,  Genbank: XM_003126990.5 | NEDD4 binding protein 1 |
| NAMPT | GeneID:595123,  Genbank: XM_021102257.1 | nicotinamide phosphoribosyltransferase |
| NEMP2 | GeneID:100515587,  Genbank: XM_021076065.1 | nuclear envelope integral membrane protein 2 |
| NEURL3 | GeneID:100524797,  Genbank: XM_003124839.5 | neuralized E3 ubiquitin protein ligase 3 |
| NLRC5 | GeneID:100135667,  Genbank: XM_021093211.1 | NLR family CARD domain containing 5 |
| NMI | GeneID:100621233,  Genbank: XM_003359414.4 | N-myc and STAT interactor |
| NOS2 | GeneID:396859,  Genbank: XM_021066186.1 | nitric oxide synthase 2 |
| NRAP | GeneID:733691,  Genbank: XM_021072190.1 | nebulin related anchoring protein |
| NUB1 | GeneID:100517250,  Genbank: XM_013990737.2 | negative regulator of ubiquitin like proteins 1 |
| NXPE3 | GeneID:100520806,  Genbank: XM_003132693.4 | neurexophilin and PC-esterase domain family member 3 |
| OAS1 | GeneID:397570,  Genbank: XM_021073680.1 | 2'-5'-oligoadenylate synthetase 1 |
| OAS2 | GeneID:595128,  Genbank: XM_021072182.1 | 2'-5'-oligoadenylate synthetase 2 |
| OASL | GeneID:595119,  Genbank: NM_001031790.1 | 2'-5'-oligoadenylate synthetase like |
| OGFR | GeneID:106508728,  Genbank: XM_021078228.1 | opioid growth factor receptor |
| OLR1 | GeneID:396724,  Genbank: NM_213805.1 | oxidized low density lipoprotein receptor 1 |
| PAQR8 | GeneID:396559,  Genbank: NM_213740.1 | progestin and adipoQ receptor family member 8 |
| PARP10 | GeneID:100516730,  Genbank: XM_003125470.6 | poly(ADP-ribose) polymerase family member 10 |
| PARP11 | GeneID:100524437,  Genbank: XM_003126559.5 | poly(ADP-ribose) polymerase family member 11 |
| PARP12 | GeneID:100515805,  Genbank: XM_003134615.4 | poly(ADP-ribose) polymerase family member 12 |
| PARP14 | GeneID:100153948,  Genbank: XM_021070260.1 | poly(ADP-ribose) polymerase family member 14 |
| PARP3 | GeneID:100620838,  Genbank: XM_013981754.2 | poly(ADP-ribose) polymerase family member 3 |
| PARP9 | GeneID:100519390,  Genbank: XM_021070264.1 | poly(ADP-ribose) polymerase family member 9 |
| PDCD1 | GeneID:100533201,  Genbank: XM_021074630.1 | programmed cell death 1 |
| PDE12 | GeneID:100157651,  Genbank: XM_001926462.5 | phosphodiesterase 12 |
| PI4K2B | GeneID:100623115,  Genbank: XM_021101073.1 | phosphatidylinositol 4-kinase type 2 beta |
| PIEZO2 | GeneID:100626687,  Genbank: XM_021096049.1 | piezo type mechanosensitive ion channel component 2 |
| PIGR | GeneID:397315,  Genbank: XM_021102216.1 | polymeric immunoglobulin receptor |
| PIK3AP1 | GeneID:100155165,  Genbank: XM_013983580.2 | phosphoinositide-3-kinase adaptor protein 1 |
| PLAC8 | GeneID:100525175,  Genbank: XM_003129352.4 | placenta specific 8 |
| PLEKHA4 | GeneID:100522871,  Genbank: XM_013998606.2 | pleckstrin homology domain containing A4 |
| PLEKHA7 | GeneID:100514820,  Genbank: XM_021083278.1 | pleckstrin homology domain containing A7 |
| PLEKHN1 | GeneID:100739230,  Genbank: XM_021095175.1 | pleckstrin homology domain containing N1 |
| PLSCR1 | GeneID:100620055,  Genbank: XM_021068460.1 | phospholipid scramblase 1 |
| PMAIP1 | GeneID:397278,  Genbank: NM_214147.2 | phorbol-12-myristate-13-acetate-induced protein 1 |
| PML | GeneID:100157336,  Genbank: XM_001925572.5 | promyelocytic leukemia |
| PPM1K | GeneID:110262190,  Genbank: XM_021101768.1 | protein phosphatase, Mg2+/Mn2+ dependent 1K |
| PPP4R4 | GeneID:100737576,  Genbank: XM_021099663.1 | protein phosphatase 4 regulatory subunit 4 |
| PRDM1 | GeneID:100154284,  Genbank: XM_005659341.3 | PR/SET domain 1 |
| PRICKLE1 | GeneID:100157209,  Genbank: XM_021092560.1 | prickle planar cell polarity protein 1 |
| PRKD2 | GeneID:100517036,  Genbank: XM_021094608.1 | protein kinase D2 |
| PROKR2 | GeneID:100625714,  Genbank: XM_003359888.4 | prokineticin receptor 2 |
| PRRG1 | GeneID:100623926,  Genbank: XM_021079580.1 | proline rich and Gla domain 1 |
| PRRG4 | GeneID:100513583,  Genbank: XM_013994445.2 | proline rich and Gla domain 4 |
| PSMB10 | GeneID:733626,  Genbank: NM_001044565.1 | proteasome subunit beta 10 |
| PSMB8 | GeneID:396975,  Genbank: NM_213935.2 | proteasome subunit beta 8 |
| PSMB9 | GeneID:654294,  Genbank: NM_001037961.1 | proteasome subunit beta 9 |
| PSME1 | GeneID:397572,  Genbank: NM_214304.1 | proteasome activator subunit 1 |
| PSME2 | GeneID:397522,  Genbank: XM_013978159.2 | proteasome activator subunit 2 |
| PSMF1 | GeneID:100152620,  Genbank: NM_001243340.1 | proteasome inhibitor subunit 1 |
| PTK2B | GeneID:100157507,  Genbank: XM_021073170.1 | protein tyrosine kinase 2 beta |
| PXK | GeneID:100515860,  Genbank: XM_005669678.3 | PX domain containing serine/threonine kinase like |
| REC8 | GeneID:100152822,  Genbank: XM_005666240.3 | REC8 meiotic recombination protein |
| RFX5 | GeneID:100517711,  Genbank: XM_003125766.4 | regulatory factor X5 |
| RGL1 | GeneID:100515847,  Genbank: XM_003357576.5 | ral guanine nucleotide dissociation stimulator like 1 |
| RHBDD1 | GeneID:100512869,  Genbank: XM_021075277.1 | rhomboid domain containing 1 |
| RNASEL | GeneID:100048946,  Genbank: NM_001097512.1 | ribonuclease L |
| RNF24 | GeneID:100519342,  Genbank: XM_021078063.1 | ring finger protein 24 |
| RSAD2 | GeneID:396752,  Genbank: NM_213817.1 | radical S-adenosyl methionine domain containing 2 |
| RTP4 | GeneID:110256385,  Genbank: XM_021070039.1 | receptor transporter protein 4 |
| S100A14 | GeneID:100153930,  Genbank: NM_001190168.1 | S100 calcium binding protein A14 |
| SDS | GeneID:100155595,  Genbank: XM_001928302.5 | serine dehydratase |
| SEMA4B | GeneID:110261555,  Genbank: XM_021098977.1 | semaphorin 4B |
| SERPING1 | GeneID:100144304,  Genbank: XM_005660873.3 | serpin family G member 1 |
| SESN2 | GeneID:100620966,  Genbank: XM_021095666.1 | sestrin 2 |
| SETX | GeneID:100511239,  Genbank: XM_021070661.1 | senataxin |
| SLA-2 | GeneID:100135031,  Genbank: XM_021097872.1 | MHC class I antigen 2 |
| SLA-7 | GeneID:396650,  Genbank: XM_021098282.1 | MHC class I antigen 7 |
| SLC15A3 | GeneID:100513135,  Genbank: XM_003122678.6 | solute carrier family 15 member 3 |
| SLC25A28 | GeneID:100526017,  Genbank: XM_003133165.4 | solute carrier family 25 member 28 |
| SLC2A12 | GeneID:100517381,  Genbank: XM_005652670.3 | solute carrier family 2 member 12 |
| SLC2A6 | GeneID:100627201,  Genbank: XM_003353701.4 | solute carrier family 2 member 6 |
| SLC41A2 | GeneID:100519603,  Genbank: XM_005664183.3 | solute carrier family 41 member 2 |
| SLC7A9 | GeneID:100037973,  Genbank: XM_021093176.1 | solute carrier family 7 member 9 |
| SLFN11 | GeneID:100625890,  Genbank: XM_021067412.1 | schlafen family member 11 |
| SMCHD1 | GeneID:100627122,  Genbank: XM_021096138.1 | structural maintenance of chromosomes flexible hinge domain containing 1 |
| SNX10 | GeneID:100520876,  Genbank: XM_021079049.1 | sorting nexin 10 |
| SOCS1 | GeneID:100307052,  Genbank: XM_005662126.3 | suppressor of cytokine signaling 1 |
| SP110 | GeneID:100519278,  Genbank: XM_021075650.1 | SP110 nuclear body protein |
| SP140 | GeneID:100516764,  Genbank: XM_021075644.1 | SP140 nuclear body protein |
| SPATA13 | GeneID:100522756,  Genbank: XM_021065472.1 | spermatogenesis associated 13 |
| SRPX2 | GeneID:100153130,  Genbank: XM_001924519.5 | sushi repeat containing protein, X-linked 2 |
| STAT1 | GeneID:396655,  Genbank: XM_021074657.1 | signal transducer and activator of transcription 1 |
| STAT2 | GeneID:396923,  Genbank: NM_213889.1 | signal transducer and activator of transcription 2 |
| STN1 | GeneID:100157838,  Genbank: XM_013983705.2 | STN1, CST complex subunit |
| TACSTD2 | GeneID:100510966,  Genbank: XM_003127967.4 | tumor associated calcium signal transducer 2 |
| TAGAP | GeneID:100155114,  Genbank: NM_001243543.1 | T-cell activation RhoGTPase activating protein |
| TAP1 | GeneID:733649,  Genbank: XM_021098370.1 | transporter 1, ATP binding cassette subfamily B member |
| TAP2 | GeneID:733650,  Genbank: XM_021098372.1 | transporter 2, ATP binding cassette subfamily B member |
| TAPBP | GeneID:100155428,  Genbank: XM_005665884.3 | TAP binding protein |
| TAPBPL | GeneID:100520908,  Genbank: XM_005653157.3 | TAP binding protein like |
| TBC1D30 | GeneID:100623817,  Genbank: XM_021091949.1 | TBC1 domain family member 30 |
| TCIRG1 | GeneID:100521769,  Genbank: XM_005660613.3 | T-cell immune regulator 1, ATPase H+ transporting V0 subunit a3 |
| TCP11L2 | GeneID:100620963,  Genbank: XM_021091578.1 | t-complex 11 like 2 |
| TDRD7 | GeneID:100152652,  Genbank: XM_001924680.5 | tudor domain containing 7 |
| TMEM106A | GeneID:100624959,  Genbank: XM_005656948.3 | transmembrane protein 106A |
| TMEM140 | GeneID:100523705,  Genbank: XM_003134650.4 | transmembrane protein 140 |
| TMEM173 | GeneID:100217389,  Genbank: XM_021077058.1 | transmembrane protein 173 |
| TMEM229B | GeneID:100153043,  Genbank: XM_005656338.3 | transmembrane protein 229B |
| TMEM268 | GeneID:100519408,  Genbank: XM_013993790.2 | transmembrane protein 268 |
| TMEM45B | GeneID:100516991,  Genbank: XM_021063152.1 | transmembrane protein 45B |
| TMTC1 | GeneID:100624475,  Genbank: XM_021092123.1 | transmembrane and tetratricopeptide repeat containing 1 |
| TNFAIP3 | GeneID:100622156,  Genbank: XM_005659182.3 | TNF alpha induced protein 3 |
| TNFSF10 | GeneID:406191,  Genbank: XM_021068572.1 | TNF superfamily member 10 |
| TNFSF13B | GeneID:100038026,  Genbank: XM_005668532.3 | TNF superfamily member 13b |
| TOR3A | GeneID:100196908,  Genbank: XM_003130340.5 | torsin family 3 member A |
| TRANK1 | GeneID:102163142,  Genbank: XM_021071655.1 | tetratricopeptide repeat and ankyrin repeat containing 1 |
| TRIM14 | GeneID:100151834,  Genbank: XM_003122031.4 | tripartite motif containing 14 |
| TRIM21 | GeneID:100302538,  Genbank: XM_005656620.3 | tripartite motif containing 21 |
| TRIM25 | GeneID:100622285,  Genbank: XM_005656971.3 | tripartite motif containing 25 |
| TRIM26 | GeneID:100144460,  Genbank: NM_001123209.1 | tripartite motif containing 26 |
| TRIM38 | GeneID:100154512,  Genbank: XM_021098422.1 | tripartite motif containing 38 |
| TRIM39 | GeneID:100151742,  Genbank: XM_013996585.2 | tripartite motif containing 39 |
| TXNIP | GeneID:733688,  Genbank: NM_001044614.2 | thioredoxin interacting protein |
| UBA7 | GeneID:100512631,  Genbank: XM_003132214.4 | ubiquitin like modifier activating enzyme 7 |
| UBD | GeneID:100294688,  Genbank: NM_001160088.1 | ubiquitin D |
| UBE2L6 | GeneID:100514753,  Genbank: NM_001246215.1 | ubiquitin conjugating enzyme E2 L6 |
| USP18 | GeneID:396777,  Genbank: NM_213826.1 | ubiquitin specific peptidase 18 |
| VWA5A | GeneID:100515913,  Genbank: XM_021063046.1 | von Willebrand factor A domain containing 5A |
| WARS | GeneID:100515390,  Genbank: XM_003128728.6 | tryptophanyl-tRNA synthetase |
| WDFY4 | GeneID:100157904,  Genbank: XM_021073024.1 | WDFY family member 4 |
| WDR76 | GeneID:100519285,  Genbank: XM_021096581.1 | WD repeat domain 76 |
| XKR9 | GeneID:100154419,  Genbank: XM_021089254.1 | XK related 9 |
| YPEL2 | GeneID:100524105,  Genbank: XM_003131686.6 | yippee like 2 |
| ZBP1 | GeneID:100144524,  Genbank: NM_001123216.1 | Z-DNA binding protein 1 |
| ZBTB5 | GeneID:110255881,  Genbank: XM_021066137.1 | zinc finger and BTB domain containing 5 |
| ZC3HAV1 | GeneID:100415778,  Genbank: XM_013990770.2 | zinc finger CCCH-type containing, antiviral 1 |
| ZCWPW1 | GeneID:100513075,  Genbank: XM_005661906.3 | zinc finger CW-type and PWWP domain containing 1 |
| ZFP36 | GeneID:100316849,  Genbank: NM_001168419.1 | ZFP36 ring finger protein |
| ZNF618 | GeneID:100157179,  Genbank: XM_021067808.1 | zinc finger protein 618 |
| ZNFX1 | GeneID:100624243,  Genbank: XM_021077518.1 | zinc finger NFX1-type containing 1 |
| ADAM28 | GeneID:100152711,  Genbank: XM_005670435.3 | ADAM metallopeptidase domain 28 |
| ANGPT1 | GeneID:397009,  Genbank: XM_021088688.1 | angiopoietin 1 |
| AREG | GeneID:397668,  Genbank: NM_214376.1 | amphiregulin |
| ARL4C | GeneID:100514255,  Genbank: NM_001244315.1 | ADP ribosylation factor like GTPase 4C |
| CD40 | GeneID:397395,  Genbank: NM_214194.1 | CD40 molecule |
| CEBPB | GeneID:397360,  Genbank: NM_001199889.1 | CCAAT/enhancer binding protein beta |
| CHAC1 | GeneID:100524723,  Genbank: XM_021084905.1 | ChaC glutathione specific gamma-glutamylcyclotransferase 1 |
| DDIT4 | GeneID:100153821,  Genbank: NM_001243452.1 | DNA damage inducible transcript 4 |
| DUSP1 | GeneID:100522469,  Genbank: XM_021076556.1 | dual specificity phosphatase 1 |
| DUSP10 | GeneID:100517416,  Genbank: XM_003130510.5 | dual specificity phosphatase 10 |
| DUSP4 | GeneID:100514374,  Genbank: XM_003133392.4 | dual specificity phosphatase 4 |
| DUSP6 | GeneID:100622968,  Genbank: NM_001267842.1 | dual specificity phosphatase 6 |
| ENPP3 | GeneID:100621671,  Genbank: XM_021087960.1 | ectonucleotide pyrophosphatase/phosphodiesterase 3 |
| EREG | GeneID:100620969,  Genbank: XM_013978775.2 | epiregulin |
| ERRFI1 | GeneID:100738544,  Genbank: XM_021095275.1 | ERBB receptor feedback inhibitor 1 |
| ETV5 | GeneID:100623208,  Genbank: XM_021068467.1 | ETS variant 5 |
| FOLR1 | GeneID:396784,  Genbank: XM_005653647.2 | folate receptor 1 (adult) |
| GDF15 | GeneID:100379630,  Genbank: NM_001174056.1 | growth differentiation factor 15 |
| HECA | GeneID:100512023,  Genbank: XM_021087304.1 | hdc homolog, cell cycle regulator |
| HES7 | GeneID:100620745,  Genbank: XM_021067858.1 | hes family bHLH transcription factor 7 |
| HFE | GeneID:110261476,  Genbank: XM_021098424.1 | hemochromatosis |
| INHBE | GeneID:100518554,  Genbank: XM_003126320.4 | inhibin beta E subunit |
| JUNB | GeneID:100523358,  Genbank: XM_005674483.3 | JunB proto-oncogene, AP-1 transcription factor subunit |
| KCNAB2 | GeneID:100514966,  Genbank: XM_021095244.1 | potassium voltage-gated channel subfamily A regulatory beta subunit 2 |
| KLF4 | GeneID:595111,  Genbank: XM_005660316.3 | Kruppel like factor 4 |
| LBH | GeneID:102160087,  Genbank: XM_021087663.1 | limb bud and heart development |
| LOC100510917 | GeneID:100510917,  Genbank: XM_003128793.4 | glutathione S-transferase A4 |
| LY6D | GeneID:100623615,  Genbank: XM_021088774.1 | lymphocyte antigen 6 family member D |
| MGAM2 | GeneID:100623494,  Genbank: XM_021079094.1 | maltase-glucoamylase 2 (putative) |
| NFATC4 | GeneID:100154469,  Genbank: XM_013978173.2 | nuclear factor of activated T-cells 4 |
| NFIL3 | GeneID:100153822,  Genbank: XM_001928870.6 | nuclear factor, interleukin 3 regulated |
| NUPR1 | GeneID:110260086,  Genbank: XM_021088038.1 | nuclear protein 1, transcriptional regulator |
| PDK2 | GeneID:100516037,  Genbank: XM_021067148.1 | pyruvate dehydrogenase kinase 2 |
| PDP1 | GeneID:100153002,  Genbank: XM_005662967.3 | pyruvate dehyrogenase phosphatase catalytic subunit 1 |
| PITX3 | GeneID:100154216,  Genbank: XM_021073065.1 | paired like homeodomain 3 |
| PLAU | GeneID:396985,  Genbank: NM_213945.1 | plasminogen activator, urokinase |
| PNRC1 | GeneID:106508885,  Genbank: XM_021089951.1 | proline rich nuclear receptor coactivator 1 |
| PPP1R1B | GeneID:100736966,  Genbank: XM_021067047.1 | protein phosphatase 1 regulatory inhibitor subunit 1B |
| RCAN1 | GeneID:100511902,  Genbank: XM_005657146.3 | regulator of calcineurin 1 |
| RHEBL1 | GeneID:100525816,  Genbank: XM_021091594.1 | Ras homolog enriched in brain like 1 |
| RNASET2 | GeneID:100157985,  Genbank: XM_001928085.5 | ribonuclease T2 |
| RND1 | GeneID:100737739,  Genbank: XM_003481567.4 | Rho family GTPase 1 |
| SAT1 | GeneID:397645,  Genbank: NM_214358.1 | spermidine/spermine N1-acetyltransferase 1 |
| SEMA4C | GeneID:100525283,  Genbank: XM_021087262.1 | semaphorin 4C |
| SERPINB1 | GeneID:100155145,  Genbank: XM_003482119.4 | serpin family B member 1 |
| SH2B3 | GeneID:100154922,  Genbank: XM_001929523.5 | SH2B adaptor protein 3 |
| SIGIRR | GeneID:100626800,  Genbank: NM_001315689.1 | single Ig and TIR domain containing |
| SLA-3 | GeneID:100037288,  Genbank: XM_021097849.1 | MHC class I antigen 3 |
| SLC2A5 | GeneID:100625876,  Genbank: XM_021095282.1 | solute carrier family 2 member 5 |
| SLC5A3 | GeneID:110255177,  Genbank: XM_005657149.3 | solute carrier family 5 member 3 |
| SLC6A9 | GeneID:100624503,  Genbank: XM_021096814.1 | solute carrier family 6 member 9 |
| SNAPC1 | GeneID:100521309,  Genbank: XM_021062485.1 | small nuclear RNA activating complex polypeptide 1 |
| SOX9 | GeneID:396840,  Genbank: NM_213843.2 | SRY-box 9 |
| SPRED3 | GeneID:100515836,  Genbank: XM_005664587.3 | sprouty related EVH1 domain containing 3 |
| TNFSF9 | GeneID:100736831,  Genbank: XM_003480815.3 | TNF superfamily member 9 |
| TRIB3 | GeneID:100518810,  Genbank: XM_013983169.2 | tribbles pseudokinase 3 |
| VEGFA | GeneID:397157,  Genbank: XM_013977975.1 | vascular endothelial growth factor A |
| YPEL5 | GeneID:100620823,  Genbank: XM_003354871.4 | yippee like 5 |
| ZFP36L1 | GeneID:100624279,  Genbank: XM_013989124.2 | ZFP36 ring finger protein like 1 |
